# Zinc Titanium Nitride Semiconductor toward Durable Photoelectrochemical Applications

**DOI:** 10.1021/jacs.2c04241

**Published:** 2022-07-20

**Authors:** Ann L. Greenaway, Sijia Ke, Theodore Culman, Kevin R. Talley, John S. Mangum, Karen N. Heinselman, Ryan S. Kingsbury, Rebecca W. Smaha, Melissa K. Gish, Elisa M. Miller, Kristin A. Persson, John M. Gregoire, Sage R. Bauers, Jeffrey B. Neaton, Adele C. Tamboli, Andriy Zakutayev

**Affiliations:** † Materials Chemical and Computational Science Directorate, National Renewable Energy Laboratory, Golden, Colorado 80401, United States; ‡ Materials and Chemical Sciences Division, Lawrence Berkeley National Laboratory, Berkeley, California 94720, United States; § Department of Materials Science and Engineering, University of California Berkeley, Berkeley, California 94720, United States; ∥ Energy Storage and Distributed Resources Division, Lawrence Berkeley National Laboratory, Berkeley, California 94720, United States; ⊥ Molecular Foundry, Lawrence Berkeley National Laboratory, Berkeley, California 94720, United States; # Division of Engineering and Applied Science, California Institute of Technology, Pasadena, California 91125, United States; ∇ Department of Physics, University of California Berkeley, Berkeley, California 94720, United States; ○ Kavli Energy Nanosciences Institute at Berkeley, Berkeley, California 94720, United States; ◆ Department of Physics, Colorado School of Mines, Golden, Colorado 80401, United States

## Abstract

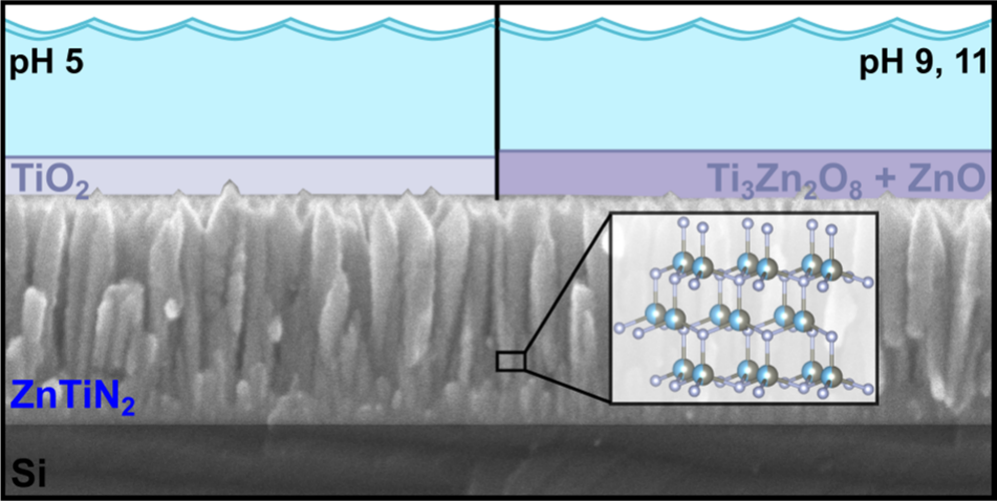

Photoelectrochemical fuel generation is a promising route to sustainable liquid fuels produced from water and captured carbon dioxide with sunlight as the energy input. Development of these technologies requires photoelectrode materials that are both photocatalytically active and operationally stable in harsh oxidative and/or reductive electrochemical environments. Such photocatalysts can be discovered based on co-design principles, wherein design for stability is based on the propensity for the photocatalyst to self-passivate under operating conditions and design for photoactivity is based on the ability to integrate the photocatalyst with established semiconductor substrates. Here, we report on the synthesis and characterization of zinc titanium nitride (ZnTiN_2_) that follows these design rules by having a wurtzite-derived crystal structure and showing self-passivating surface oxides created by electrochemical polarization. The sputtered ZnTiN_2_ thin films have optical absorption onsets below 2 eV and n-type electrical conduction of 3 S/cm. The band gap of this material is reduced from the 3.36 eV theoretical value by cation-site disorder, and the impact of cation antisites on the band structure of ZnTiN_2_ is explored using density functional theory. Under electrochemical polarization, the ZnTiN_2_ surfaces have TiO_2_- or ZnO-like character, consistent with Materials Project Pourbaix calculations predicting the formation of stable solid phases under near-neutral pH. These results show that ZnTiN_2_ is a promising candidate for photoelectrochemical liquid fuel generation and demonstrate a new materials design approach to other photoelectrodes with self-passivating native operational surface chemistry.

## 1 Introduction

Photoelectrochemical carbon dioxide reduction (PEC CO_2_R) is a promising route to recycling captured CO_2_ in the form of liquid chemical fuels using sunlight as the energy input. Practical PEC CO_2_R systems will require the development of multiple components, including high-performing catalysts,^1^ separation membranes,^2^ and coupled microenvironments,^3,4^ as well as the integration and scale-up of these materials and processes.^5^ A critical factor for the future success of PEC CO_2_R is the identification of suitable photoelectrode materials to convert photons to electrons to drive CO_2_R as well as the oxygen evolution reaction (OER) that enables the production of fuel using only CO_2_ and H_2_O as reactants.^6,7^ Like semiconductors used in photovoltaics, these photoabsorbers are subject to stringent requirements of strong absorptivity, long carrier lifetimes, and appropriate band gap, but with the added constraints of band edge positions that can drive the reaction of interest, suitable adsorption/desorption kinetics at the surface, and, critically, operational stability in aqueous environments.^6,8^

Photocatalyst discovery campaigns have focused on down-selecting from a broad range of candidate materials based on thermodynamic and electronic structure criteria, with great success in identifying photocatalysts that meet some but not all performance requirements.^6,9−11^ The primary challenge framed by prior work is the simultaneous realization of long-term operational stability and high radiative efficiency. This grand challenge can be addressed by a co-design approach, wherein photocatalyst design originates from device-level experiential knowledge. Recent implementations in other technologies have demonstrated the value of co-design for solving challenging multiobjective problems.^12,13^ In the present case, we design photocatalysts based on experience from photoelectrochemical and photovoltaic devices. From photoelectrochemical devices, we embrace electrochemical self-passivation, focusing on kinetic (as opposed to thermodynamic) operational stabilization. From photovoltaics, we recognize that synthetic control of defects is paramount to rapid translation from materials discovery to high-efficiency devices and that heteroepitaxial growth on established semiconductor substrates is a demonstrated method to enable high-efficiency absorbers. A key attribute of co-design is disruption of the sequential design process, which for photocatalysts has traditionally been materials discovery based on performance criteria followed by synthesis for device implementation. Co-design ensures that integration and scale-up processes accompany each discovered photocatalyst, a transformational research approach that can amplify the impact of discovery science.^14^

While the most-investigated photovoltaic materials (Si, III–Vs, and II–VI compounds) have promising properties for PEC applications, all corrode rapidly under electrochemical operation, a result of their low Pourbaix stability.^7,15−17^ Substantial efforts have been dedicated to limiting such corrosion,^18,19^ but these have failed to generate photoelectrodes with surfaces that are durable for more than tens of hours of operation. While these issues can be partially addressed via the application of a protection layer, such approaches are undesirable due to complex processing^20^ and the propensity for degradation from electrolyte infiltration at pinholes or grain boundaries.^15^ The lesson from prior device implementations is that even with protective coatings, the semiconductor light absorber must self-passivate under operating conditions. Though metal oxides have been a common target of photoanode searches due in part to their relatively small driving force for corrosion compared to traditional photovoltaic semiconductors,^9−11^ the most prolific solar energy converter, BiVO_4_, suffers from its lack of self-passivation,^21,22^ and new classes of self-passivating oxides such as copper vanadates^23^ suffer from poor carrier transport. Searches beyond metal oxides have identified promising candidates that do self-passivate, such as Ta_3_N_5_^24−26^ and Sb_2_S_3_,^27−29^ but further development has been hampered by the inability to effectively integrate these semiconductors into high-efficiency photoelectrochemical generators.

In photoelectrode co-design, it is critical to consider materials that could be paired with established semiconductors to impart good material quality *via* heteroepitaxy and have surfaces that transform under operation in aqueous conditions to stable coatings with compatible crystal structures. Recent work in computational materials discovery has predicted a trove of nitride semiconductors with earth-abundant constituent elements that merit evaluation against these criteria.^30,31^ A family of Zn- or Mg-based multivalent ternaries with crystal structures derived from wurtzite or rocksalt parent compounds^32,33^ is particularly promising; these nitrides can be integrated with wide-band-gap GaN and related III-N wurtzite semiconductors that are amenable to p-type doping for contact formation. Examples of the experimentally synthesized wurtzite materials in this family include Zn_2_VN_3_,^34^ MgSnN_2_,^35,36^ Zn_2_NbN_3_,^37^ Zn_3_MoN_4_,^38^ Zn_2_SbN_3_,^39^ Mg_2_SbN_3_,^40^ Mg_2_PN_3_, and Zn_2_PN_3_,^41,42^ among others. For several well-studied materials in this family, such as wurtzite ZnSnN_2_^43,44^ and ZnGeN_2_^45−47^ as well as rocksalt Mg_2_NbN_3_^48^ and MgZrN_2_,^49−51^^49−51^ elemental disorder on the cation sublattice of the parent structure has been shown to influence both band gap and transport properties,^45,52^ although this phenomenon has not been studied across the broader class.^52−54^ However, none of the above multivalent ternary nitrides have been considered for PEC applications, with research in this space limited to wurtzite oxynitride alloys such as ZnGeN_2_-ZnO^55^ or ZnSnN_2_-ZnO,^56^ despite the potential for integration with GaN (or other III-N),^57,58^ and many other theoretical predictions have never been synthesized so their experimental properties remain unknown.

One particularly suitable candidate material in this chemical and structural space is ZnTiN_2_, which has not previously been synthesized. This material has been theoretically predicted to be stable with a cation-ordered wurtzite-derived crystal structure compatible with wurtzite GaN,^59^ with other independent computational studies supporting these theoretical predictions.^30,31^ Materials Project Pourbaix stability calculations^7,60−62^ indicate that ZnTiN_2_ will decompose to stable oxides such as ZnO and TiO_2_ in near-neutral pH aqueous environments. These ZnO and TiO_2_ decomposition products are not only electrochemically stable under the operating conditions but are also utilized in other applications as transparent conducting oxides with good electrical charge transport and wide optical band gaps. In addition, TiO_2_ has been extensively studied as an archetype photoelectrochemical fuel generation material with exceptional stability^7^ and as a stabilizing coating layer with suitable charge transport properties on high-quality Si and III–V photovoltaic absorbers used in photoelectrochemical applications.^19^ The known phenomenon of band-gap reduction from cation-site disorder introduces an additional aspect of co-design for this material. Co-design based on realized, rather than idealized, properties has been leveraged in industrial applications;^63^ here, we anticipated that cation-disordered ZnTiN_2_ would have a band gap reduced from the previously computed 3.5 eV, bringing the band gap into the range needed for photoelectrochemical fuel-forming applications. All of these theoretical predictions and scientific hypotheses together call for experimental investigations of ZnTiN_2_.

Here we report on the synthesis and characterization of zinc titanium nitride (ZnTiN_2_) and evaluate its chemical and physical properties toward photoelectrochemical applications. The sputtered thin films crystallize in cation-disordered wurtzite-derived structure with strong (002) preferential orientation normal to the substrate surface, in a composition window from stoichiometric ZnTiN_2_ up to ∼60% Zn on the cation site, and with unintentional oxygen incorporation of less than 10 anion % in the bulk of the layers. The ZnTiN_2_ films show an optical absorption onset close to 2 eV from both optical absorption and transient absorption spectroscopy, and n-type transport with high electron doping indicated by 0.3 Ω-cm electrical resistivity and *S* = −50 μV K^–1^ Seebeck coefficient. Density functional theory (DFT) calculations show that this reduced band gap compared to the previously predicted 3.5 eV^31^ value may be due to band shifts caused by nonstoichiometric N-centered Zn_1_Ti_3_ and Zn_3_Ti_1_ tetrahedral motifs in cation-disordered ZnTiN_2_, the presence of which broadens the band structure near the band edges, reducing the gap. ZnTiN_2_ electrodes show ZnO-like or TiO_2_-like character depending on the pH operating conditions and regardless of the applied potential near CO_2_R and OER operating conditions. These results show that the ZnTiN_2_ wurtzite semiconductor may have bulk optoelectronic properties and self-passivating surface chemistry suitable for photoelectrochemical fuel generation and point to a new material design strategy for photoelectrode development.

## 2 Results and Discussion

### 2.1 Crystal Structure and Phase Competition

**Figure 1:**
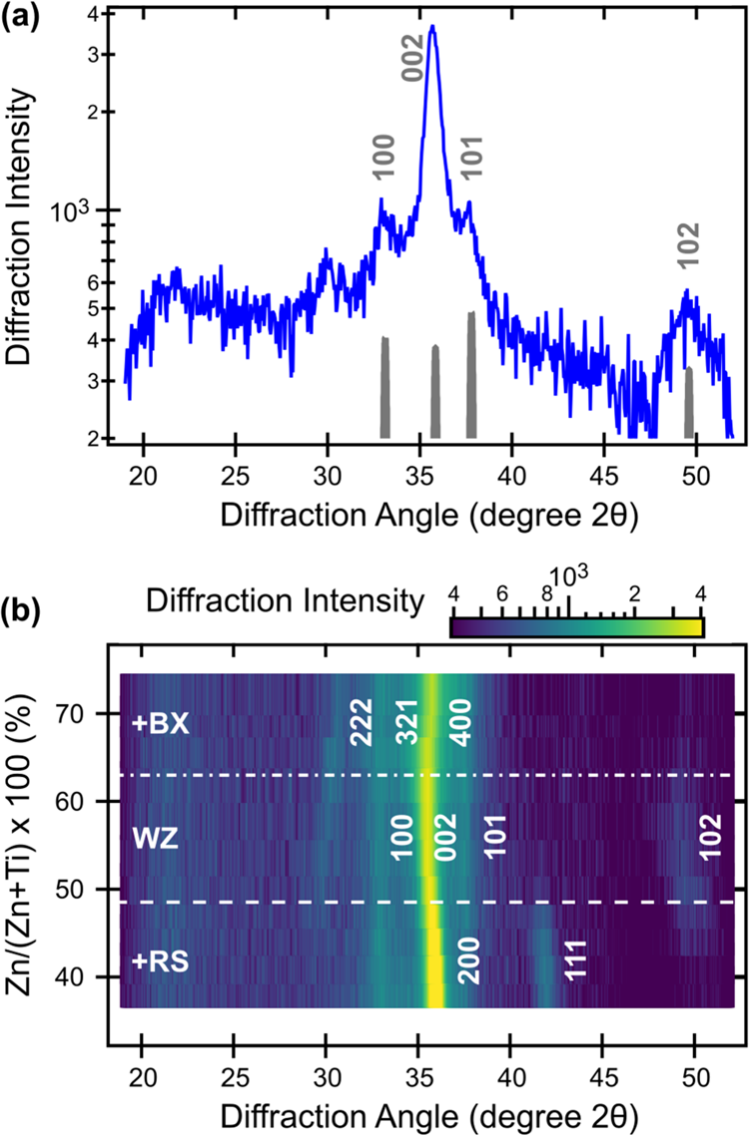
X-ray diffraction (XRD, Cu Kα radiation) of polycrystalline ZnTiN_2_ thin films. (a) Diffraction from a near-stoichiometric (Zn/(Zn + Ti) = 0.5) polycrystalline film, with an experiment-modeled disordered ZnTiN_2_ diffraction pattern (gray, refer to description in text). (b) Composition-dependent XRD heat map illustrating the presence of other phases at low (RS) and high (BX) Zn/(Zn + Ti).

Synthesis of ZnTiN_2_ films was carried out using radio-frequency reactive co-sputtering (see 4. Experimental Section). Figure 1 shows the results of X-ray diffraction (XRD) measurements of ZnTiN_2_ thin films with varying cation composition, Zn/(Zn + Ti), measured by X-ray fluorescence (XRF). The XRD patterns of these polycrystalline samples deposited at ambient temperature on Si substrates have the strongest peak at 2θ = 36°, as well as several weaker reflections. As shown in Figure 1a for the ZnTiN_2_ sample with Zn/(Zn + Ti) = 0.5, this main peak can be attributed to the (002) wurtzite (WZ) reflection, as supported by the (100) peak at 33°, (101) peak at 38°, and (102) peak at 49°. Computational work predicts an orthorhombic structure (*Pna*2_1_, space group 33) for cation-ordered ZnTiN_2_;^30,31,59^ like other ternary nitrides of this type,^35,44,47,52^ ZnTiN_2_ experimentally takes a cation-disordered, wurtzite-type structure. The absence of low-angle reflections (between 20 and 25° 2θ) and high-angle peak splitting are indicative of the long-range cation disorder in this material.^52^ Assuming an orthorhombic unit cell, the lattice constants deduced from the experimental peak positions are *a* = 5.4 Å, *b* = 6.2 Å, *c* = 5.0 Å; for a wurtzite-type structure, the lattice constants would be *a* = 3.1 Å and *c* = 5.0 Å. The strong intensity of the (002) peak indicates that the ZnTiN_2_ films have strong *c*-axis preferential orientation, which has been reported for other ternary nitrides with a wurtzite-derived crystal structure.^44,47^

The WZ-derived structure is stable from 0.5 < Zn/(Zn + Ti) < 0.6, where the high-angle WZ (102) peak at 49° coexists with a minor secondary phase with a peak at 30°, as shown in Figure 1b. For the Ti-rich (Zn-poor) compositions of Zn/(Zn + Ti) < 0.5, there is a clear secondary rocksalt-derived (RS) phase, likely TiN, indicated by the (111) peak at 42°, as well as strengthening and a clear shift of the highest intensity peak toward higher angle to become RS TiN (200). For the Zn-rich (Ti-poor) compositions, Zn/(Zn + Ti) > 0.5, there is a secondary fluorite-derived phase, likely anti-bixbyite (BX) Zn_3_N_2_; this assignment is supported by the (222) reflection at 30° accompanied by a small shift of the main (400) peak to a higher angle. Annealing these materials in N_2_ atmosphere did not substantially change the crystallinity or phase content up to 500–600 °C and led to rapid Zn, N, and thickness loss at and above 700 °C (see Figure S1 in the Supporting Information (SI)). The finite composition width of the WZ phase space, as well as the competition with the RS phase (on the Zn-poor side) and BX phase (on the Zn-rich side), are similar to what has been reported for other zinc transition-metal nitrides.^34,64^

**Figure 2:**
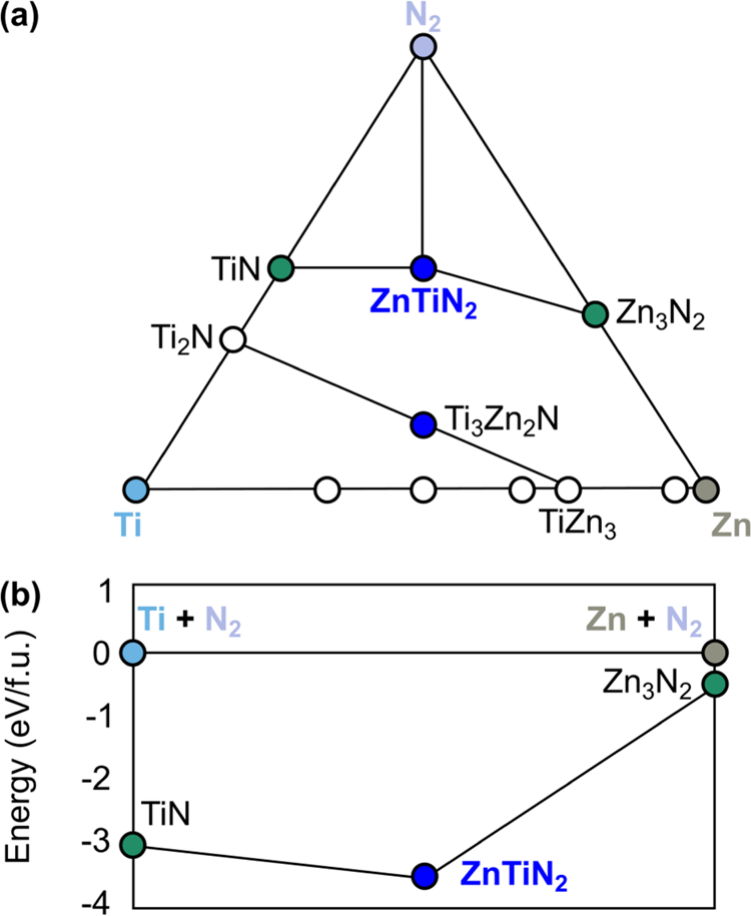
Calculated thermochemical stability of ZnTiN_2_: (a) three-dimensional (3D) convex hull adapted from ref (30); (b) two-dimensional (2D) convex hull projection based on calculations from the NRELMatDB, ref (65), consistent with the calculations in ref (30). Blue circles represent stable ternary materials, green circles represent stable binary compounds, and white circles are unstable compounds. Elemental reference states are given with associated colors.

The experimental observation of ZnTiN_2_ with a WZ-derived structure (Figure 1a) and its competing RS and BX phases (Figure 1b) are generally consistent with the previously published theoretical predictions for crystal structure and thermodynamic stability of this material. Figure 2 shows the convex hull stability diagrams for the full Zn–Ti–N ternary space (Figure 2a) and along a pseudo-binary (Zn_3_N_2_)-(TiN + N_2_) tie-line (Figure 2b), adapted from ref (30). According to these DFT calculations, the main competing phases of ZnTiN_2_ are Zn_3_N_2_ on the Zn-rich side and TiN on the Ti-rich side of the ternary composition, in agreement with our experimental measurements (Figure 1b). The calculated formation enthalpy of ZnTiN_2_ from the elements was −1.154 eV/atom, and its decomposition energy into the competing Zn_3_N_2_ and TiN phases was +0.035 eV/atom,^30^ indicating ZnTiN_2_ is a thermodynamically stable material. It is important to note that these theoretical predictions were made for cation-ordered ZnTiN_2_, whereas experimental measurements do not show evidence of such long-range ordering (Figure 1). However, as shown below (Section 2.4), the computed energy to interchange Zn and Ti atoms in ZnTiN_2_, introducing antisite defects and off-stoichiometric motifs that reduce the band gap, can be as small as ∼0.01 eV/atom depending on concentration and the specific configuration. This is significantly smaller than the calculated formation enthalpies or decomposition energies discussed here.

### 2.2 Synthetic Control of Crystallinity in (002)-Oriented Films

**Figure 3:**
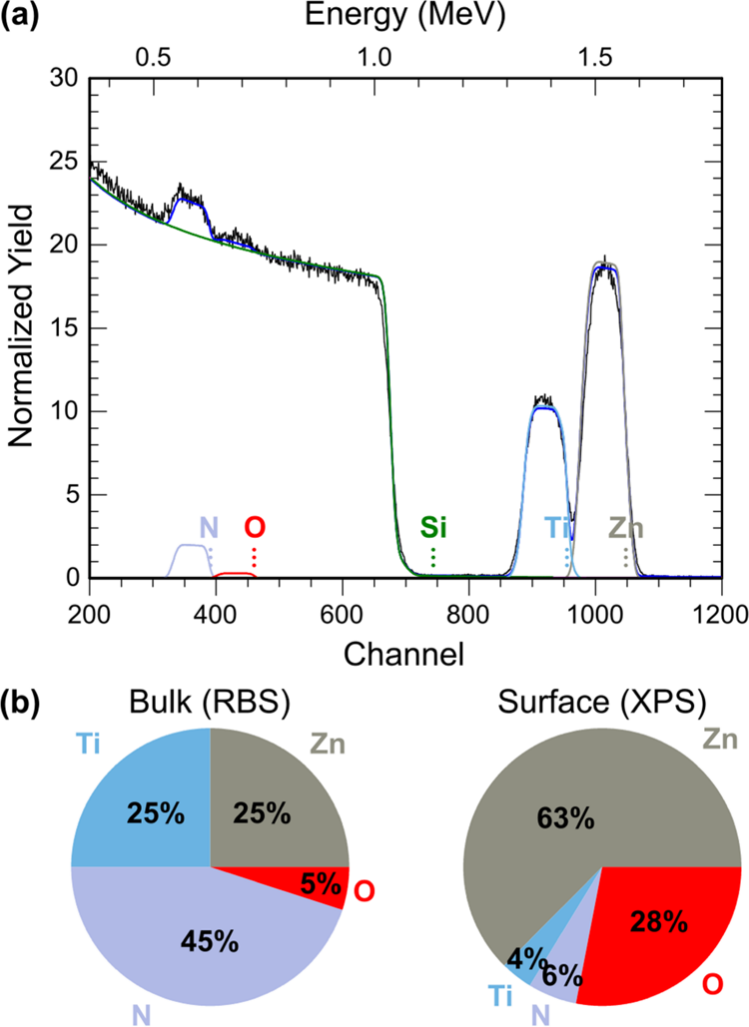
Composition of ZnTiN_2_ thin films. (a) Representative RBS spectrum showing contributions from Zn, Ti, N, O, and the substrate (Si). The overall fit is shown as a solid blue line. (b) Pie charts comparing bulk (RBS, from (a)) and surface (top ∼10 nm, XPS) compositions of representative films.

Rutherford backscattering spectrometry (RBS) data were acquired from a film with Zn/(Zn + Ti) ≈ 0.5 as measured by XRF (Figure 3a). A two-layer model of the Si substrate and the ZnTiN_2_ film constrained to equal numbers of cations and anions indicates equal amounts of Zn and Ti, in agreement with XRF. The RBS data also show the presence of oxygen, approximately 10% of the total anion composition, O/(N + O). Some fraction of this oxygen is likely due to oxidation of films after removal from the deposition chamber. Figure 3b compares the bulk composition of the film (from RBS) with the surface composition (from X-ray photoelectron spectroscopy, XPS), which shows that the film is heavily oxidized and substantially Zn-rich at the surface. A negative Seebeck coefficient measured from these films (discussed later) does, however, suggest the incorporation of some n-type O impurities on N sites (O_N_) in the film.

**Figure 4:**
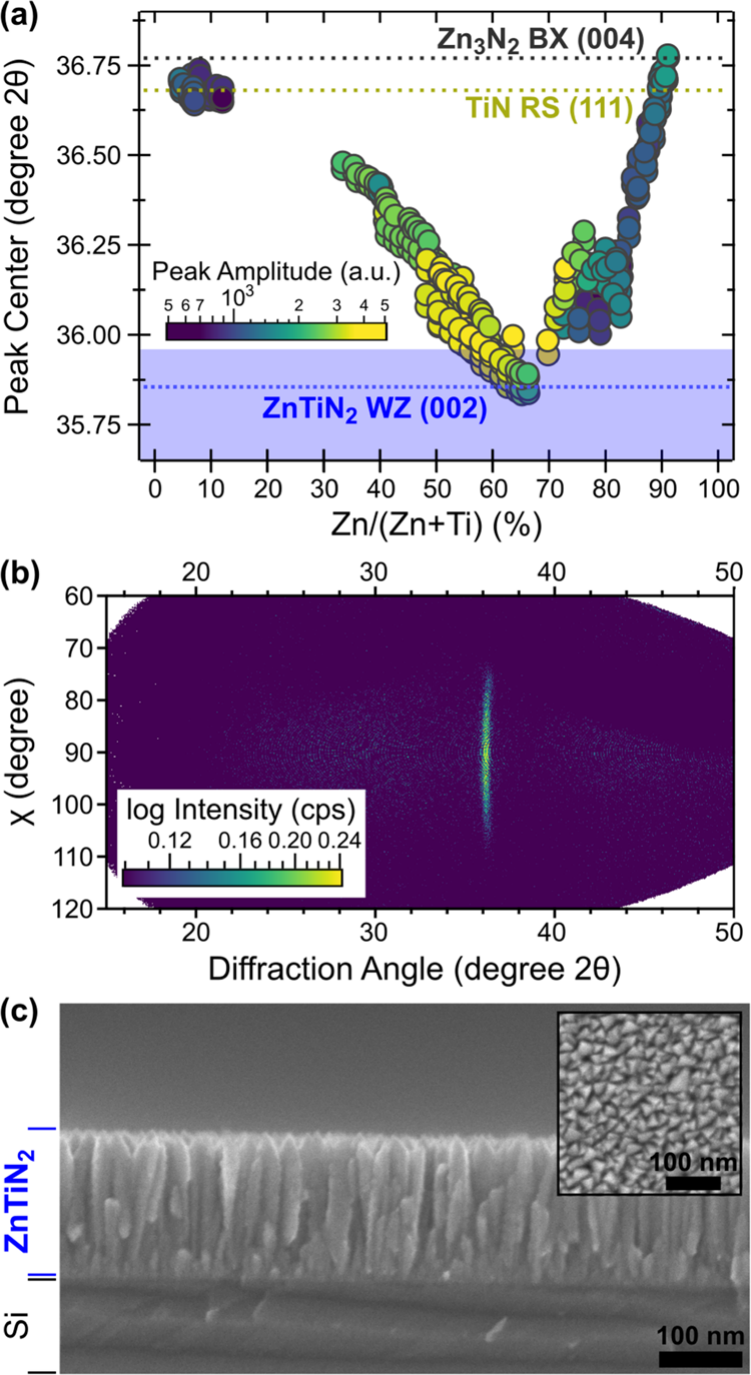
Crystallinity of ZnTiN_2_ thin films. (a) Peak position vs composition for an exploration of deposition conditions, with peak amplitude used as a proxy for crystallinity. Peak positions are from binary nitride phases (Zn_3_N_2_: ICSD #84918,^66^ TiN: ICSD #656836^67^) or, for ZnTiN_2_, highlighted as a range based on the experimental peak position shift for the (002) peak of the WZ phase in Figure 1b. Deposition conditions for the ZnTiN_2_ films presented here are given in Table S1. (b) Representative area detector signal (Cu Kα radiation) for optimized ZnTiN_2_ deposition with strong (002) texturing, showing a single peak at 2θ = 36° with a 40° width in χ. Grazing-incidence XRD confirming the presence of wurtzite signature peaks can be seen in Figure S2. (c) Cross-sectional SEM showing columnar grains and textured morphology with plan-view inset showing triangular cross sections; both scale bars are 100 nm.

To improve the crystallinity of the ZnTiN_2_ material and study its optical and electrical properties, highly textured (002)-oriented films were grown by RF co-sputtering directly onto heated EXG glass substrates by a single-step deposition process (complete deposition conditions are given in Table S1). Factors influencing crystallinity were studied by tracking the position and amplitude of the diffraction reflection around 2θ = 36° as a function of composition and deposition temperature (Figure 4a). The cation composition of the films ranged from 0.05 < Zn/(Zn + Ti) < 0.91 and was primarily controlled by modifying the relative powers of the Ti and Zn sputtering targets. Less Zn incorporation was observed as the deposition temperature set point (for films with temperature gradients, described in 4. Experimental Section and the SIs) was increased from 175 to 310 °C while keeping the target powers constant, due to the higher volatility of Zn compared to Ti. Around 0.5 < Zn/(Zn + Ti) < 0.6, the peak amplitude is maximized. The peak center is closest to that of the (002) reflection of the wurtzite ZnTiN_2_ structure at slightly higher Zn compositions, 0.6 < Zn/(Zn + Ti) < 0.65. As the film cation composition becomes either Ti-rich or Zn-rich, the peak amplitude decreases and the peak center shifts to a higher 2θ, consistent with the additional phases observed in Figure 1b. For films with near-stoichiometric cation compositions, Zn/(Zn + Ti) = 0.5, a single reflection, shown in Figure 4b, is observed in 2D X-ray diffraction centered around χ = 90° and 2θ ≈ 36°, corresponding to the (002) reflection of WZ ZnTiN_2_. The spread in χ from approximately 70 to 110° clearly displays the textured nature of the films, in agreement with the tilted columnar microstructure observed by cross-sectional scanning electron microscope (SEM) (Figure 4c). The nominal film thickness, measured by SEM, is ∼150 nm. Most of the textured columnar microstructure exhibits grain sizes ranging from 20 to 30 nm in the horizontal in-plane direction and from 50 to 150 nm in the vertical growth direction. However, in the first 20 nm of growth, the microstructure consists of much smaller grains, which could be due to different nucleation and growth conditions in the early stages of film deposition. As observed in both cross-sectional and plan-view (Figure 4c, inset) images, the film surface is rough due to the rounded tops of the columnar grains.

### 2.3 Optoelectronic Properties of Cation-Disordered ZnTiN_2_

**Figure 5:**
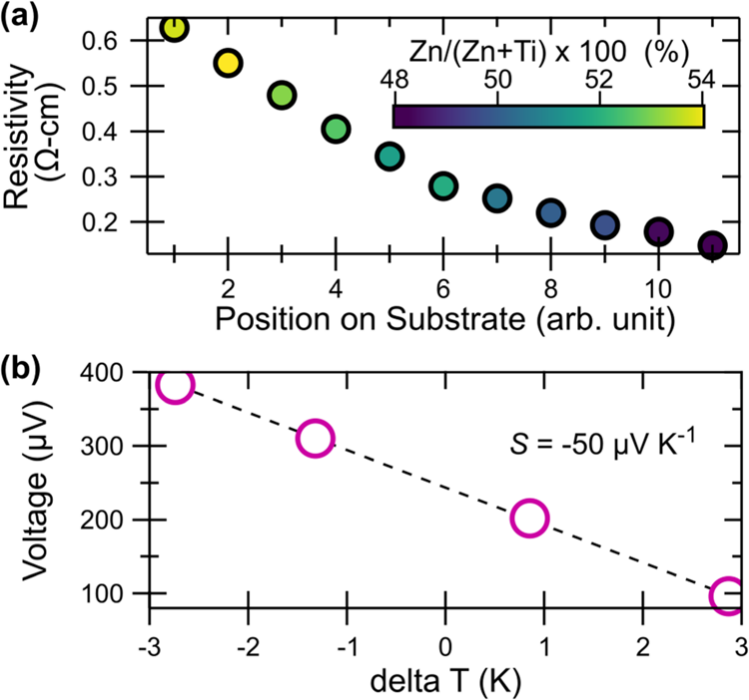
Electrical properties of cation-disordered ZnTiN_2_ (a) Resistivity across cation composition, Zn/(Zn + Ti) extracted from four-point probe measurements. The *x* axis indicates the position on the substrate and illustrates the even nature of the cation gradient across the substrate. (b) Seebeck characteristics, where the circles are measured data and the dotted line is the fit.

Electronic transport measurements were carried out to determine the carrier dynamics of sputtered ZnTiN_2_ thin films. Colinear four-point probe resistivity measurements collected on films with near-stoichiometric compositions, 0.48 < Zn/(Zn + Ti) < 0.54, show a resistivity of ca. ρ = 0.3 Ω-cm (σ = 3 S cm^–1^) with Ti-rich concentrations having a lower resistivity (Figure 5a), as expected with the addition of electrons into the conduction band of an already n-type material. To verify that ZnTiN_2_ is n-type, we performed near-room-temperature Seebeck coefficient measurements and found *S* = −50 μV K^–1^ (Figure 5b). The negative sign confirms electrons as the majority carrier and the magnitude is indicative of a highly doped semiconductor. Hall measurements from stoichiometric ZnTiN_2_ were unsuccessful due to low in-plane mobility, which is not unexpected due to the combination of the columnar textured morphology and percent-scale oxygen impurity (see Figure 3a). In-plane transport measurements are expected to exhibit low charge carrier mobility due to grain boundary scattering from the columnar microstructure, which is often seen in sputtered thin films (see Figure 4c). Assuming an upper limit on carrier mobility of μ = 0.1 cm^2^ V^–1^ s^–1^ along with the fundamental relationship σ = *ne*μ, we estimate a lower bound on the carrier density of *n* ≈ 10^19^ cm^–3^. Carrier densities of this order of magnitude are commonly observed in early-stage nitride semiconductors and arise primarily from oxygen impurities. Since O concentrations are on the several % scale (see Figure 3a) and each O_N_ defect has an excess valence electron, even small dopant activation efficiencies will lead to large carrier densities.

**Figure 6:**
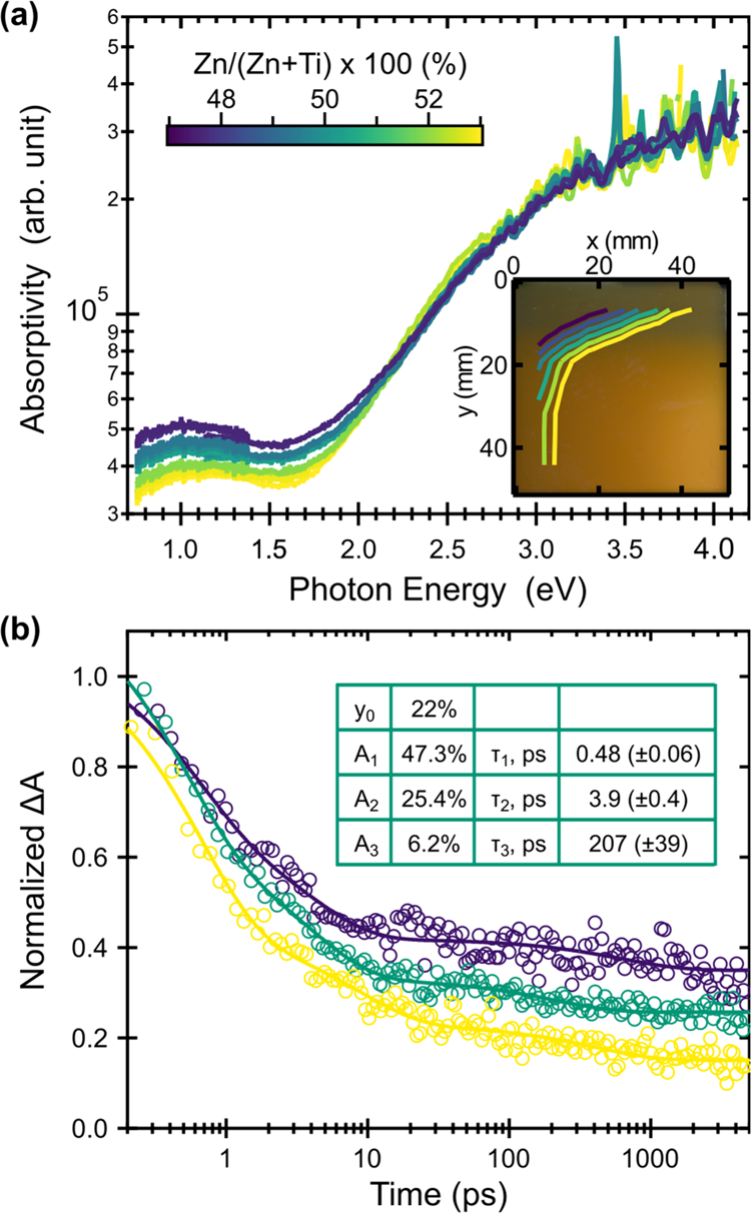
Optical properties of cation-disordered ZnTiN_2_. (a) Absorptivity, with the inset showing cation composition contours across a photograph of a ZnTiN_2_ sample. The cation composition scale bar applies to the entire figure. (b) Normalized transient absorption kinetics at a probe energy of 2.07 eV at varying Zn/(Zn + Ti) = 47% (purple), 50% (teal), and 53% (yellow) after 3.1 eV photoexcitation. The inset table displays the results of the fit at Zn/(Zn + Ti) = 50%. Additional fit data are shown in Table S2; the full transient absorption of Zn/(Zn + Ti) = 50% and a comparison of the three films’ response at 0.25 ps is shown in Figure S4.

The optical properties of ZnTiN_2_ thin films with 0.46 < Zn/(Zn + Ti) < 0.53 were studied using ultraviolet–visible–near-infrared (UV–vis–NIR) transmission and reflection spectroscopy, and absorptivity was calculated (Figure 6a). For all compositions, a drop in the reflection-corrected transmission (Figure S3) is observed near 2 eV, as expected from a mid-gap semiconductor similar to other II-IV-N_2_ materials.^32,47^ Conversion to absorptivity shows an onset in the 1.5–2.0 eV range, with a slightly lower-energy onset for films with high Zn concentration. While this trend may point to a degree of band gap tunability with composition, the signal may alternatively arise from a Burstein–Moss shift rather than an increase in the fundamental gap as the excess electrons arising from antisite Ti_Zn_ defects would populate the conduction band. There is less shift in the absorption curve for Zn-rich films, though alloys of ternary nitride wurtzite semiconductors and ZnO are also known to affect optical absorption properties.^68,69^ These data indicate some subgap absorption, likely a result of the high carrier concentration of the ZnTiN_2_ films.^70^ In all cases, 1.5–2.0 eV is significantly lower than the ∼3.5 eV band gap previously predicted for ZnTiN_2_, which is likely related to the cation disorder as observed in XRD.

Photoexcitation of ZnTiN_2_ thin films with a 3.1 eV pump pulse produces a broad photoinduced absorption in the visible regime that undergoes rapid relaxation with the majority of carriers in all films lost within the first picosecond (Figure 6b). This behavior is similar to photoelectrode materials β-Mn_2_V_2_O_7_^71^ and BiVO_4_^72^ early in their development. For the film with Zn/(Zn + Ti) = 50%, kinetics are consistent throughout the spectral window with a 0.48 ps time constant accounting for 47% of the loss of signal. Longer components of 3.9 ps and 207 ps are responsible for 25 and 6.2% of the decay, respectively. There is a small (22%) long-lived signal that persists beyond our 5 ns time window. This behavior is consistent with photoexcitation above the band gap followed by a rapid loss of approximately 78% of the photogenerated free carriers. The off-stoichiometric films (Zn/(Zn + Ti) = 47 and 53%) show similar behavior, with the Zn-rich film having shorter lifetimes, consistent with an increase in recombination pathways supported by increased resistivity from Figure 5a. Kinetics for the off-stoichiometric films are shown in Table S2. Figure S4 shows transient absorption spectra for Zn/(Zn + Ti) = 50% as well as a comparison of the 0.25 ps spectra of all three films, which corroborates the shift in absorption onset from higher to lower energy with increasing Zn/(Zn + Ti) observed in Figure 6a.

### 2.4 Electronic Structure of ZnTiN_2_

**Figure 7:**
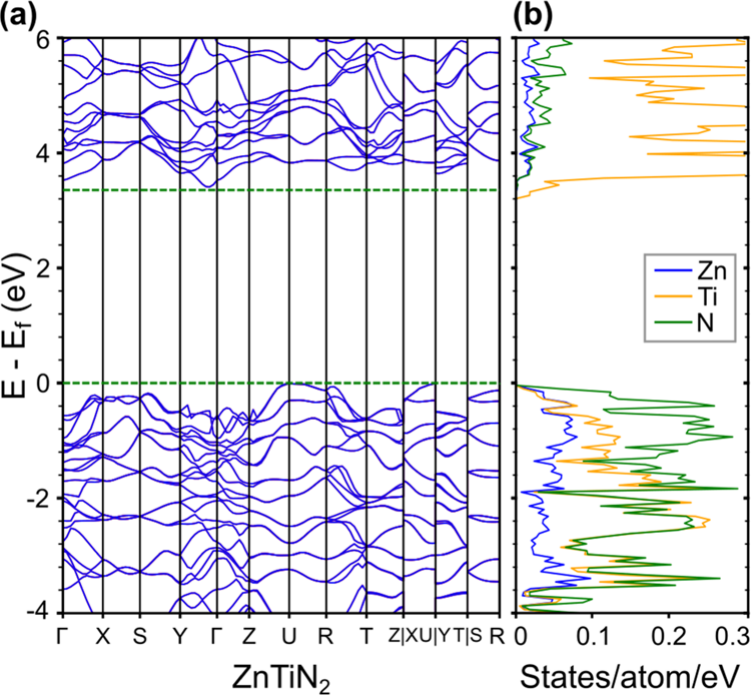
(a) Calculated electronic band structure with DFT-HSE of cation-ordered ZnTiN_2_. (b) Projected DOS.

To understand the difference between the predicted 3.5 eV band gap of cation-ordered ZnTiN_2_ previously reported^31^ and the experimentally measured optical absorption onset reported here, we performed DFT calculations introducing specific types of cation disorder (see 4. Experimental Section). Cation-ordered ZnTiN_2_ takes up an orthorhombic structure (*Pna*2_1_, space group 33) containing four formula units, and can be thought of as a crystalline network of corner-sharing N-centered tetrahedra with two Zn and two Ti at the vertices, denoted as N–Zn_2_Ti_2_. DFT-Perdew–Burke–Ernzerhof (PBE) calculated lattice parameters are *a* = 5.71 Å, *b* = 6.59 Å, and *c* = 5.26 Å, slightly overestimating the experimentally derived parameters (Section 2.1). N-cation bond lengths for the N–Zn_2_Ti_2_ motif (and for the other motifs discussed later) are shown in Table 1. Figure 7 shows the band structure of cation-ordered ZnTiN_2_, calculated with DFT-HSE. Our computed DFT-HSE band gap is 3.36 eV, which is consistent with previous calculations^31^ but overestimates our experimental measurements (our DFT-PBE band gap is significantly smaller at 2.25 eV, as expected). While DFT-PBE is expected to underestimate the band gap, DFT-HSE is known to improve the accuracy of the gap for some materials.^73^ Examining the projected density of states (DOS) for the cation-ordered structure, we find that the valence band edges are dominated by N p states, consistent with reports of other zinc ternary nitrides with similar structure, such as ZnSnN_2_^31,74,75^ and ZnGeN_2_.^54,76^ The conduction band edges are dominated by Ti d states, in contrast to ZnSnN_2_ and ZnGeN_2_ where conduction band edges are dominated by N p states.^31,74,75^

**Table 1:** Calculated N-Cation Bond Lengths for the Three Types of Tetrahedral Motifs

	N–Zn_2_Ti_2_	N–Zn_1_Ti_3_	N–Zn_3_Ti_1_
N–Zn bond length (Å)	2.06	2.14	2.02
N–Ti bond length (Å)	1.95	1.99	1.87

**Figure 8:**
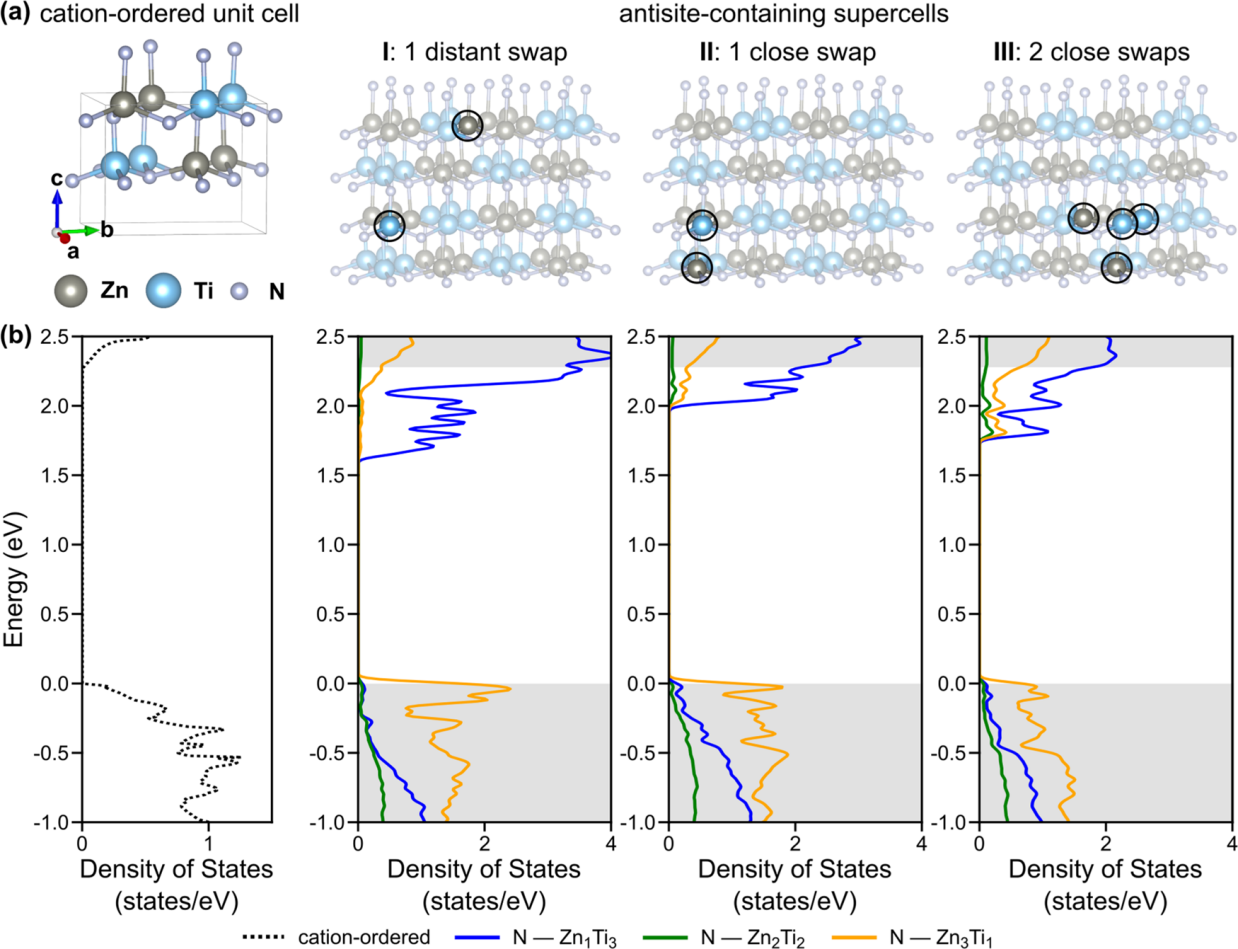
(a) Crystal structures of cation-ordered ZnTiN_2_ and three antisite-containing 128-atom supercells with the exchanged Zn and Ti atoms highlighted. (b) Partial density of states per N-centered tetrahedra for the cation-ordered material and the three supercells, with the contributions from the different N-centered tetrahedra illustrated. Gray shading indicates the band edges from the cation-ordered material. Gaussian smearing is used in our Brillouin zone integrations, using a smearing parameter of 0.03 eV.

The experimentally synthesized ZnTiN_2_ is cation-disordered by XRD; that is, the material features a high density of antisite defects, where the positions of Zn and Ti atoms are swapped relative to the cation-ordered structure (although experimental quantification of cation-site disorder is difficult, see ref (52)). To computationally investigate the influence of these antisite defects on the electronic structure, we construct 2 × 2 × 2 supercells containing 32 formula units (128 atoms) and introduce one or more Zn–Ti antisite defects. The primary motif of the cation-ordered structure, an N-centered Zn_2_Ti_2_ tetrahedron, obeys the octet rule, where each N-centered tetrahedron has exactly two Zn and two Ti atoms such that charge neutrality is conserved locally.^77^ Swapping a Zn and Ti atom in the structure introduces two types of octet-rule-violating N-centered tetrahedra, N–Zn_1_Ti_3_ and N–Zn_3_Ti_1_. We focus on three supercells in which we introduce N–Zn_1_Ti_3_ and N–Zn_3_Ti_1_ tetrahedra to investigate different examples of cation disorder. In supercell I, we swap a single Zn and Ti between distant (non-neighboring) tetrahedra; in supercell II, we swap a single Zn and Ti in one tetrahedron; in supercell III, we perform two swaps of Zn and Ti inside one N-centered tetrahedron. The numbers of octet-violating tetrahedra introduced in each supercell are shown in Table 2, and the supercells are shown in Figure 8a, with changes to N-cation bond lengths reported in Table 1. We compute the energetic cost of creating these antisite defects to be on the order of tens of meV per formula unit, comparable to prior work on cation-disordered ZnGeN_2_^52,76^ and consistent with the observation of cation disorder in ZnTiN_2_ deposited at ambient temperatures. We note that while the specific cation swaps considered here do not necessarily reflect the most likely atomic configurations,^74,78^ they nonetheless provide insight into the impact of antisite defects on the electronic structure of ZnTiN_2_, as we show below.

**Table 2:** Calculated Band Gaps and Energy Costs of ZnTiN_2_ with Different Antisite Types from 128-Atom Supercells^a^

	cation-ordered ZnTiN_2_	I: 1 distant swap	II: 1 close swap	III: 2 close swaps
N–Zn_1_Ti_3_ motif density	0	6.25% (4/64)	4.69% (3/64)	9.38% (6/64)
N–Zn_3_Ti_1_ motif density	0	6.25%	4.69%	9.38%
band gap (eV)	2.25	1.64	2.00	1.78
relative energy per formula unit (eV)	0	0.042	0.024	0.049

a All tabulated band gaps and energies are computed with DFT-PBE.

Our calculations of the electronic structure of our antisite-containing supercells demonstrate that the presence of antisite defects significantly reduces the band gap relative to the cation-ordered structures for all three supercells (Table 2), although the orbital character of the band edges does not change. The DFT-PBE band gap in supercell I is computed to be reduced by 0.61 eV and the gap in supercell II is reduced by 0.25 eV. Relative to our computed DFT-PBE gap of the cation-ordered phase of 2.25 eV, these represent significant reductions of 11 and 27%, respectively. Introducing more octet-rule-violating N-centered tetrahedra in supercell III, with two close swaps, we find that the DFT-PBE band gap of ZnTiN_2_ is reduced by 0.47 eV (21% relative to the cation-ordered reference), intermediate between the other two supercells. The band gap reductions we computed in these three supercells are consistent with previous calculations that consider antisite defects in ZnGeN_2_^54^ and ZnSnN_2_,^74^ providing qualitative trends to describe cation disorder although the number and distribution of defects will likely be higher in real material. From these calculations, we can conclude that the band gap reduction difference is affected by both the number of the octet-rule-violating tetrahedra and their relative spatial separation. The smaller band gap reduction in the supercell III with 2 close swaps may result from the clustering of octet-rule-violating N-centered tetrahedra, and partial compensation by neighboring tetrahedra.

To better understand the calculated reduction in band gap with antisite defects, we examine the contributions of each of the three types of tetrahedra (N–Zn_1_Ti_3_, N–Zn_3_Ti_1_, and N–Zn_2_Ti_2_) to the projected partial DOS (from DFT-PBE) for each antisite-containing supercell and compare to the cation-ordered case (Figure 8b). In all three supercells, states closer to the conduction band edges are dominated by N–Zn_1_Ti_3_ tetrahedra, and states closer to the valence band edges are dominated by N–Zn_3_Ti_1_. For N–Zn_1_Ti_3_, the conduction band edge states are predominately of Ti character; for N–Zn_3_Ti_1_, the valence band edge states are predominantly of N character. Since the corner atoms of the octet-rule-violating N–Zn_1_Ti_3_ tetrahedra would be more positively charged than in N–Zn_2_Ti_2_, intuitively the binding energy of electrons is increased relative to N–Zn_2_Ti_2_, introducing states below the cation-ordered conduction band edge and resulting of an overall downward shift of the N–Zn_1_Ti_3_ corresponding bands toward lower energies relative to the cation-ordered structure. Along the same lines, the corner atoms of N–Zn_3_Ti_1_ would be less positively charged, decreasing the electron binding energy and introducing states above the valence band edge, leading to an upward shift of the N–Zn_3_Ti_1_ corresponding bands to higher energies. In this way, the contributions to the electronic structure from both types of tetrahedra associated with cation disorder lead to an overall reduction in band gap. We note that the presence of these antisite defects not only leads to a shift in energy of the conduction and valence band edges (reducing the gap), but it also increases the localization of states in conduction band edges and valence band edges, as evidenced by the reduced band dispersion.

### 2.5 Electrochemical and Surface Properties

**Figure 9:**
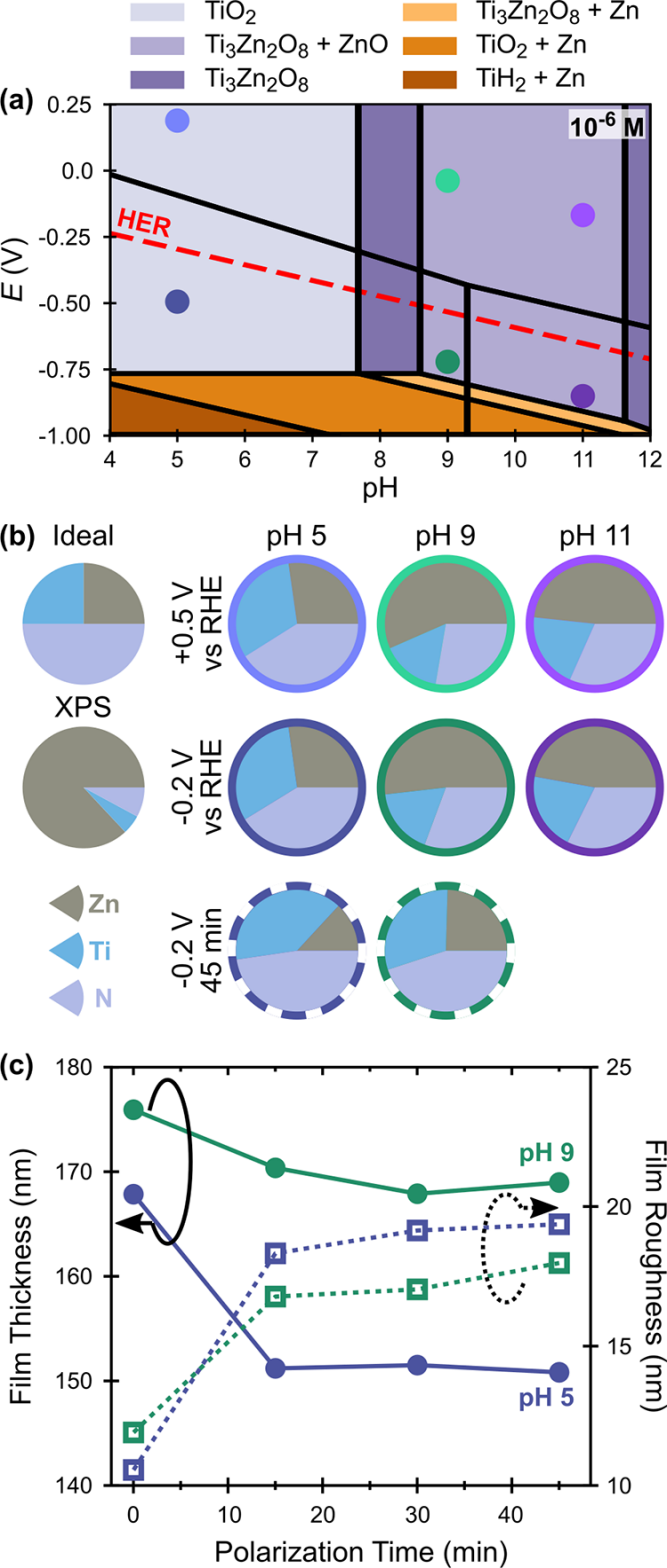
(a) Calculated Pourbaix diagram for ZnTiN_2_ at 10^–6^ M from the Materials Project, showing only stable solid phases. See Figure S5 for complete diagrams. Round markers in (a) correlate to (b), where pie charts illustrate the surface composition of the ZnTiN_2_ films (by XPS, normalized to Zn, Ti, N) following polarization for 15 min at either +0.5 or −0.2 V vs RHE, and for 45 min at −0.2 V vs RHE (dashed outlines), in comparison to the ideal composition and the previously measured surface composition by XPS (Figure 3b). Oxygen is omitted due to the convoluting presence of O-containing supporting electrolytes. Full elemental characterization of the surfaces, including residual supporting electrolyte, can be seen in Table S3. (c) Changes in ZnTiN_2_ film thickness by spectroscopic ellipsometry during extended polarization (45 min) at −0.2 V vs RHE in pH 5 (blue) and pH 9 (green). Error bars for thickness and roughness measurements are too small to resolve on the scale of the figure; at pH 5, thickness is ±0.4 nm and roughness is ±0.2 nm; at pH 9, thickness is ±0.1 nm and roughness is ±0.05 nm.

With an understanding of the synthesis and materials chemistry of ZnTiN_2_, we investigate the behavior of this material under CO_2_R-relevant conditions. Figure 9a shows a selected Pourbaix region for ZnTiN_2_ calculated using data from the Materials Project^60,61^ (complete diagrams with multiple ionic concentrations are given in Figure S5). These diagrams are built from a combination of r^2^SCAN meta generalized gradient approximation (GGA) and PBE GGA DFT calculations using the computational Pourbaix formalism of Persson et al.^61^ and the DFT mixing scheme of Kingsbury et al.^62^ (see 4. Experimental Section). Including metaGGA calculations is beneficial because SCAN (on which r^2^SCAN is based) was shown to predict ternary nitride formation enthalpies in the nitrogen-rich region of the phase diagram more accurately than GGA.^79^ At the near-neutral, reducing conditions required for CO_2_R, the ZnTiN_2_ surface is expected to decompose to either TiO_2_ or Ti_3_Zn_2_O_8_ with or without ZnO, depending on the solution concentrations of Zn, Ti, and N. Both TiO_2_^19^ and ZnO^80^ are established protective coatings for photoelectrodes; thus, the calculations suggest that ZnTiN_2_ may be stabilizable as a photoabsorber for CO_2_R. The Pourbaix analysis indicates that these same passivation layers may stabilize ZnTiN_2_ under OER conditions over the entire pH range (see Figure S5), highlighting the breadth of opportunities for its further development as a solar photocatalyst.

To further evaluate passivation of the ZnTiN_2_, nominally cation stoichiometric ZnTiN_2_ alloys (0.48 < Zn/(Zn + Ti) < 0.52) were electrochemically polarized in the dark to either −0.2 or +0.5 V vs the reversible hydrogen electrode (RHE) for 15 min at pH 5, 9, or 11. As discussed above (see Figure 3b), XPS shows that the initial film surface (∼top 10 nm) is very Zn-rich and has oxidized substantially, with very little N present. Changes to the surface compositions are shown in Figure 9b; these changes correlate more strongly with pH than *E*, as surface compositions are roughly the same by pH. At pH 5, concentrations of N are near-stoichiometric (∼41% of the total Zn + Ti + N), and there is slightly more Ti than Zn present. At pH 9 and 11, the surface is ∼50% Zn, and substantially more Ti and N are observed than in the XPS measurement of the unpolarized surface. Only the concentrations of Zn, Ti, and N are compared to track changes to these bulk elements at the surface. Oxygen concentrations are omitted for surface compositions of the polarized samples due to the convoluting presence of O-containing supporting electrolytes (phosphate and carbonate).^7^ Full surface elemental compositions can be found in Table S3. Two additional electrodes were polarized at −0.2 V vs RHE at pH 5 and 9 for a total of 45 min to confirm that the ZnTiN_2_ films were not continuously dissolving (Figure 9c). For both films, there was an initial thinning and roughening of the film (determined by modeling spectroscopic ellipsometry data) which did not continue after the 15 min timepoint. The surface compositions of these films are also shown in Figure 9b and are consistent with both the 15 min compositions and Pourbaix calculations.

**Figure 10:**
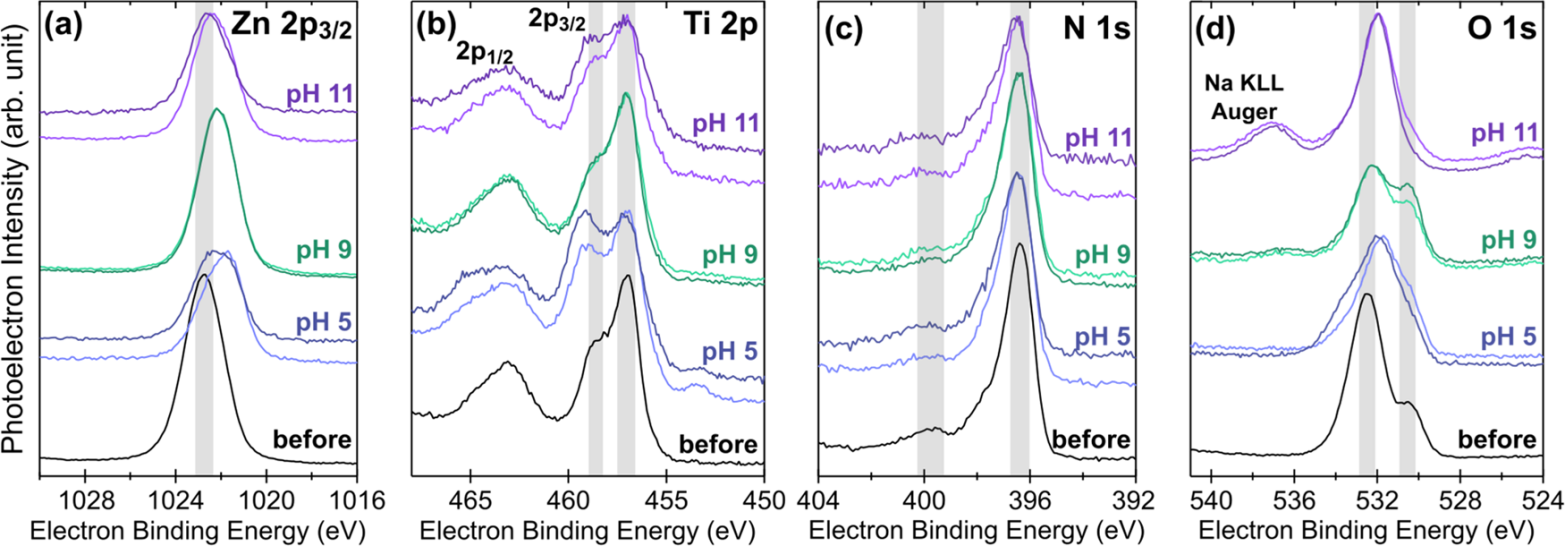
XPS spectra for individual elements comparing the initial ZnTiN_2_ surface (before) from a representative film to the results of the 15 min polarization studies (labeled with pH; darker color indicates −0.2 V, while lighter color indicates +0.5 V polarization, as in Figure 9). Gray bars are a guide to the eye for indicative peaks described in the text. (a) Zn 2p_3/2_, (b) Ti 2p, (c) N 1s, and (d) O 1s. XPS spectra for the 45 min polarizations are given in Figure S7.

XPS spectra for each element across 15 min polarization conditions are compared to the representative film before polarization in Figure 10. The Zn 2p_3/2_ spectra strongly indicate only one oxidation state across conditions (consistent with Zn^2+^ in both ZnO and ZnTiN_2_).^81^ In contrast, there are multiple environments for Ti^4+^, with relative amounts changing with pH. We attribute the lower-energy peak at ∼457 eV to the bulk environment of ZnTiN_2_ and the higher-energy peak at ∼458 eV to the formation of TiO_2_.^82^ No clear indication of TiN (∼456 eV) is present, but there may be a small contribution at pH 11; at pH 5, there is a small amount of reduced Ti^0^ (∼454 eV). The N 1s spectra indicate N^3–^ and change only minimally with polarization at any condition, consistent with N leaving the film rather than changing oxidation state at the surface. This is in turn consistent with the Materials Project Pourbaix calculations which indicate no stable solid phases incorporating N. Finally, the O 1s spectra initially show two distinct bonding environments, which we attribute to metal oxides (∼530.5 eV) and ZnO with O vacancies (∼532.5 eV),^83^ consistent with the pre-polarization surface stoichiometry. After polarization, the O spectra are complicated by the retention of supporting electrolyte, which contributes substantially to the observed intensity. Residual phosphates at pH 5 and 11 dominate the spectra, while at pH 9, the higher-energy peak can be attributed to carbonate.^84^

The changes in composition and XPS spectra with polarization indicate the evolution of the ZnTiN_2_ surface, although XRD collected on the films show no new crystalline phases in the bulk of the material (Figure S6). The surface changes at pH 5 are distinct from those at pH 9 and 11, which are broadly similar. After polarization at pH 5, the surface compositions are closer to the RBS-measured bulk film composition than the pre-polarization XPS composition (see Figure 3), indicating the loss of the initial ZnO-rich surface, which is not stable at this pH. The near-stoichiometric N concentration indicates that if a surface oxide is present after polarization, that oxide is not thicker than the probe depth of the XPS (∼10 nm). The peak assigned to TiO_2_ in the Ti 2p XPS spectra is enhanced in both pH 5 scans compared to the pre-polarization surface, suggesting TiO_2_ formation. The peaks in the Zn 2p_3/2_ and O 1s spectra are also shifted, which also support the removal of the pre-polarization ZnO and, with the N concentration, exposure of ZnTiN_2_ at the surface. This is consistent with the Materials Project Pourbaix calculations which show that ZnO should not be stable at pH 5 regardless of solution concentration, while TiO_2_ is stable (Figures 9a and S4). After 45 min, the film surface has nearly 40% Ti present, consistent with the continued loss of Zn in favor of TiO_2_ formation.

After polarizations at pH 9 and 11, there is more N present than in the pre-polarization scan (but less than at the surface at pH 5). The peaks in the Zn 2p_3/2_ spectra are closely aligned with their pre-polarization position, as are the O 1s peaks at ∼532.5 eV, suggesting that the pre-polarization ZnO environment has not changed dramatically. The Materials Project Pourbaix calculations indicate that the surface composition at these pH values is largely concentration-dependent, with a mixed Ti_3_Zn_2_O_8_ + ZnO occurring at higher solution ion concentrations and TiO_2_ at lower concentrations (Figure 9a–c). The increased concentration of Ti at the surface and changes to the Ti 2p spectra at pH 11 (increase in the ∼458 eV peak relative to the ∼456 eV peak) may indicate the formation of some Ti-containing oxide, but because the ZnTiN_2_ surface was initially very Zn-rich, the stoichiometric ratio of Zn:Ti cannot be used to identify the formation of the mixed oxide phase. However, these data do suggest that a thin oxide persists at the surface of the ZnTiN_2_ film at alkaline pH. Although 45 min of polarization in pH 9 results in a slightly Ti-rich surface, the XPS spectra of this film are qualitatively similar to the 15 min polarization (Figure S7). With the data indicating TiO_2_ formation at the surface at pH 5, these are promising indicators for further investigation of ZnTiN_2_ as a CO_2_R or OER photoelectrode across aqueous environments.

## 3 Summary and Conclusions

Herein we envision a new generation of photocatalysts discovered through co-design for operational stability and facile integration into high-efficiency devices. We report the first photocatalyst identified via this co-design approach, ZnTiN_2_. We investigate the crystal structure and physical properties of ZnTiN_2_ synthesized by reactive sputtering from metallic Zn and Ti precursors in a N_2_ atmosphere. We investigate the optoelectronic properties, including photoresponse, and the surface chemistry of these thin films under electrochemical conditions to evaluate them for potential (photo)electrochemical applications, such as in CO_2_R and OER. Overall, the newly synthesized ZnTiN_2_ wurtzite semiconductor may have bulk optoelectronic properties and self-passivating surface chemistry suitable for photoelectrochemical fuel generation.

The sputtered ZnTiN_2_ thin films with columnar microstructure form in a cation-disordered wurtzite-derived crystal structure with strong (002) preferential orientation, in a relatively broad range of Zn-rich compositions limited by the formation of rocksalt TiN and anti-bixbyite Zn_3_N_2_. Chemical composition measurements indicate unintentional oxygen incorporation of less than 10% of the anion content in the bulk of the layers, with self-passivating zinc-based native oxide formation at the film surface. The ZnTiN_2_ films show 0.3 Ω-cm electrical resistivity and *S* = −50 μV K^–1^ Seebeck coefficient, indicating n-type conduction and suggesting high electron doping; the measured photoresponse of the films is consistent with this high doping level. The measured optical absorption onset of these cation-disordered films is close to 2 eV, which is significantly lower than the 3.36 eV theoretical band gap for cation-ordered ZnTiN_2_ determined here by N 2p-derived valence band maximum and Ti 3d-derived conduction band minimum. Theoretical calculations reported here show that the difference is attributable to band gap narrowing due to the upward shift of the valence band caused by N-centered Zn_3_Ti_1_ tetrahedral motifs, and downward conduction band shift caused by the N–Zn_1_Ti_3_ motifs in cation-disordered ZnTiN_2_. XPS measurements indicate that the ZnTiN_2_ photoelectrode surfaces exposed to high pH (9, 11) have ZnO-like character, whereas the pH 5 treated surfaces show some TiO_2_-like character as well as exposed ZnTiN_2_, regardless of the applied potential in the studied range.

To realize the full potential of ZnTiN_2_ as a semiconductor for photoelectrochemical applications, it would be important to improve its charge transport properties by growing high-quality thin films on lattice-matched substrates. Epitaxial growth on p-type GaN would be particularly promising for improving the photoexcited hole extraction from the n-type ZnTiN_2_ absorber. Another critical step will be to evaluate potential epitaxial relations of ZnTiN_2_ with the ZnO and TiO_2_ decomposition products and study band alignment for charge transport between this absorber and its self-passivating surface coatings. The results reported in this paper, as well as the future research directions discussed here, illustrate a new materials design strategy to develop photoelectrochemically active semiconductors with native operational surface chemistry tuned for durability under their operating conditions.

## 4 Experimental Section

### 4.1 Synthesis

Initial polycrystalline Zn–Ti–N films were deposited by co-sputtering from Zn and Ti targets in N plasma on stationary (001)-oriented Si substrates without intentional heating, in a custom vacuum chamber with a base pressure of <10^–7^ Torr enhanced by a cryoshroud surrounding the plasma zone. The 2″ diameter targets were pointed at an angle at the substrate and were excited by radio-frequency (RF) field of Zn = 13 W and Ti = 60 W. The N plasma intensity was enhanced by RF plasma source at 350 W. The deposition pressure was set to 6 mTorr by flowing 3 sccm of N_2_ and 6 sccm of Ar through a partially closed gate valve. The 1-h deposition led to a 50 nm thick Zn–Ti–N film, with a gradient of cation composition across the sample resulting from the stationary substrate and angled targets during the deposition. Before and after the Zn–Ti–N deposition, 40 nm thick AlN layers were deposited by sputtering from a metallic Al target at 60 W onto a rotating substrate under the same conditions to enhance polycrystallinity of the resulting material for structure identification, and to suppress Zn volatilization during annealing. These samples were subject to rapid thermal annealing (RTA) in flowing N_2_ atmosphere at ambient pressure for 3 min in *T* = 500–700 °C temperature range, following a 3 min hold at 100 °C to drive off water.

Optimization of Zn–Ti–N film crystallinity was performed in a second custom vacuum chamber, with a base pressure of <10^–7^ Torr enhanced by a cryoshroud surrounding the plasma zone. Highly textured Zn–Ti–N films were deposited by co-sputtering from 2″ Zn and Ti targets in a N and Ar plasma onto stationary Corning EXG glass substrates; the targets were positioned 180 degrees from one another and pointed at an angle toward the substrate, creating a composition gradient in the deposited film. Target powers were varied between Zn = 10 to 25 W and Ti = 100 to 150 W to tune the range of the composition gradient. The deposition pressure was either 6 or 3.5 mTorr with flows of 50 sccm of N_2_ and 100 sccm of Ar. A calibrated temperature gradient was introduced perpendicular to the composition gradient by heating one end of the substrate and allowing the thermal diffusivity of the EXG glass to create a gradient in temperature (see SI). Using the knowledge gained from optimizing crystallinity, some highly textured, near-stoichiometric films were deposited at ambient temperature onto p-type single-crystal silicon or EXG glass substrates by co-sputtering from Ti (100 W) and ZnTi alloy (150 W) 2″ targets for specific use in electrical, optical, microscopy characterizations as well as electrochemical studies. All depositions were conducted for 2 h following substrate temperature stabilization and 30 min of pre-sputtering with the substrate covered by a shutter.

### 4.2 Characterization

Cation composition, reported as the ratio of Zn/(Zn + Ti), was measured by collecting and analyzing X-ray fluorescence spectra (XRF) with a Fischer XDV-SDD and the accompanying analysis software. X-ray diffraction (XRD) was acquired over a range of 2θ = 19–52° and χ = 60–120° using a Bruker D8 Discover equipped with an area detector. Scanning electron microscopy was performed using a Hitachi S-4800 operating at 3 kV accelerating voltage, 5 μA emission current, and a working distance of 3.5 mm. Rutherford backscattering spectrometry (RBS) data were acquired using a Model 3S-MR10 RBS system from National Electrostatics Corporation. The RBS measurements were performed using 2 MeV α particles in a 168° backscatter configuration for a total accumulated charge of 80 μC, and compositions were determined by modeling the spectra using RUMP software using the simplest possible two-layer (substrate + film) model.^85^ Anion concentrations were visually overfit by the software’s built-in least-squares algorithm, likely related to a weak signal for low-Z elements on top of a large substrate background. Instead, we modeled various fixed anion combinations and found a good qualitative match when [N] + [O] = 2 (i.e., equal to [Zn] + [Ti] for stoichiometric compositions) and [O]/([O] + [N]) = 0.1. Mapping data (XRD, XRF, UV–vis, and four-point probe) were analyzed using CombIgor, a custom *Igor Pro* (WaveMetrics, Lake Oswego, OR) add-on.^86^ Data were harvested and processed using NREL’s Research Data Infrastrcture^87^ and will be made available through the High Throughput Experimental Materials Database.^88^

Optical data were collected on a custom-built UV–vis/NIR transmission and reflection optical spectroscopy instrument equipped with halogen and deuterium lamps with a 0.75–4.2 eV range. The transmission and reflection spectra were used to calculate absorptivity, α = −ln[*T*/(1 – *R*)]/*t*, where *t* is the thickness as measured by stylus profilometry (Dektak). The as-measured high α values (ca. 10^5^ cm^–1^) are likely due to optical losses from imperfections in the Au and Al mirrors used as reflectance standards, so α values are reported in arbitrary units. The slight discontinuity in the overlapping spectral regions is due to differences in experimental imperfections when moving from NIR to UV–vis optical setups. Library photographs were collected using a color-calibrated desktop scanner in transmission mode. Transient absorption data were collected using a Coherent Libra Ti:sapphire laser (1 kHz, 800 nm (1.55 eV) fundamental, 150 fs pulse width). The 3.1 eV (400 nm, 500 nJ/pulse) pump pulse was generated in a TOPAS-C optical parametric amplifier and the white light probe pulses were produced via supercontinuum generation in a thin sapphire window (λ_probe_ = 2.8–1.55 eV). A mechanical delay stage was used to delay the probe relative to the pump and pump and probe were spatially overlapped at the sample. A portion of the probe was picked off before the sample to reduce noise to <0.1 mOD. A fiber-optic coupled multichannel spectrometer with a CMOS sensor was used to monitor changes in the probe. Helios software from Ultrafast Systems was used to collect the data and the data were chirp corrected and analyzed with Ultrafast Systems’ SurfaceXplorer software.

Resistivity data were collected on a colinear four-point probe instrument by sweeping current between the outer two pins while measuring the voltage between the inner pins. Conventional geometric corrections were applied to convert the measured resistance into sheet resistance and then resistivity. Seebeck coefficients were measured on a lab-built instrument. Here, the sample is suspended across two thermally and electrically isolated copper blocks, each fitted with cartridge heaters and embedded copper-constantan thermocouples, with contacts made by pressed indium dots. One block is heated to slightly above room temperature while the temperature of each is monitored by the thermocouples and the thermovoltage is measured by the like-metal thermocouple leads across two blocks. This is repeated to create a d*V*/d*T* curve, the slope of which is then corrected for the instrument’s calibrated Seebeck coefficient to determine the sample Seebeck coefficient.

Electrochemical polarization experiments used ZnTiN_2_ films deposited on conductive Si with 0.48 < Zn/(Zn + Ti) < 0.52. XPS data for the as-grown samples were obtained on an Omicron XPS setup and were consistent across the composition range. Films were fabricated into electrodes using electrodeposition tape and acted as working electrodes with a Pt counter electrode and saturated calomel reference electrode. Electrolytes were buffered to the correct pH (5, 9, or 11) using Hydrion buffers. ZnTiN_2_ electrodes were polarized using a BioLogic SP-300 at either −0.2 or +0.5 V vs RHE (corrected for pH) for 15 min. For extended polarization studies, electrodes were polarized for 15 min intervals, removed from the electrochemical cell and dried, characterized by spectroscopic ellipsometry, and then returned to the solution for further polarization. Ellipsometry measurements were conducted with a J.A. Woollam Co. M-2000 variable angle spectroscopic ellipsometer over a wavelength range of 300–1700 nm at angles of 65°, 70°, and 75°. The raw data were analyzed using the CompleteEase software by modeling the films with a generalized oscillator model consisting of one Tauc-Lorentz and two Gaussian oscillators to account for the semi-absorbing nature of the films. Film thickness and surface roughness were determined by fitting the model to the raw data with a mean squared error less than 20. Post electrochemical polarization, XPS data were obtained on a Physical Electronics Versa Probe III using Al Kα radiation. For both pre- and postpolarization XPS measurements, the XPS setup was calibrated with Au and/or Cu metal, which was cleaned via Ar-ion sputtering. The raw atomic concentration has a 5% error due to surface inhomogeneities, surface roughness, literature sensitivity values for peak integration, *etc*.

### 4.3 Calculations

Our density functional theory (DFT) calculations within the generalized gradient approximation (GGA) are performed with the Vienna Ab Initio Simulation Package (VASP).^89,90^ We use the exchange-correlation functional of Perdew, Burke, and Ernzerhof (PBE)^91^ to relax structures and compute electronic structures. In certain cases, we also use the Heyd–Scuseria–Ernzerhof (HSE06) screened hybrid functional^92,93^ to compute electronic structure. An energy cutoff of 600 eV is used for all calculations. Total energies are converged to within 10^–5^ eV and all Hellmann–Feynman forces are below 0.01 eV/Å on each atom. For all calculations, we use the projector augmented wave potentials, treating 3d2 4s2, 3d10 4s2, and 2s2 2p3 electrons explicitly for Ti, Zn, and N, respectively. We consider cation-ordered and -disordered structures. The cation-ordered orthorhombic structure (16 atoms) has a *Pna*2_1_ space group and contains 4 formula units (f.u.). Our cation-disordered structure (128 atoms) is based on a 2 × 2 × 2 supercell of the cation-ordered structure but includes selected antisite defects. A Γ-centered 10 × 10 × 8 Monkhorst–Pack *k*-mesh is used for calculations involving the 4 f.u. cation-ordered unit cell, and a 4 × 4 × 4 *k*-mesh is used in 16 f.u. supercell. Gaussian smearing is used in our Brillouin zone integrations, using a smearing parameter of 0.02 eV in structure relaxation and 0.03 eV in static density of states calculation. The N–Ti or N–Zn bond lengths in the octet-rule-violating N-centered tetrahedra motif in Table 1 are the averaged bond lengths in each cation-disordered supercell structure, which is the same within 0.01 Å among three supercells, while N–Ti or N–Zn bond length in N–Zn_2_Ti_2_ is the averaged bond length in cation-ordered structure.

Our electrochemical stability calculations (Pourbaix diagrams) are built from a combination of PBE GGA DFT calculations retrieved from the Materials Project database and regulated-restored strongly constrained and appropriately normed (r^2^SCAN) metaGGA calculations calculated using the workflow detailed in Kingsbury et al.^94^ We used the Materials Project DFT mixing scheme^62^ to combine these two sets of calculations and create a solid phase diagram of the Zn–N–Ti–O–H chemical system, from which we constructed Pourbaix diagrams using the computational formalism of Persson et al.^61^ The mixing scheme allowed us to build the convex energy hull from higher-level metaGGA calculations by recomputing only the stable phases and phases close to the hull with r^2^SCAN (170 calculations total) instead of the entire Zn–N–Ti–O–H chemical system (more than 500 total phases according to the Materials Project Database). For two stable phases where the large number of sites made r^2^SCAN structure optimizations impractical (Ti_3_Zn_22_ and Ti_20_H_2_N_17_, with 100 and 39 sites, respectively) we employed single-point calculations, as suggested in Kingsbury et al.^62^ The stability predictions of this mixed Pourbaix diagram were qualitatively similar to those obtained from a pure GGA phase diagram constructed without any r^2^SCAN calculations, but predicted a slightly larger region of decomposition to Ti_3_Zn_2_O_8_ and/or ZnO (see Figure S3).

## Supporting Information Available

Details of annealing studies of ZnTiN_2_ thin films, deposition parameters, grazing-incidence X-ray diffraction on highly (002) textured films, UV–vis–NIR reflection-corrected transmission, transient absorption spectroscopy, calculated Pourbaix diagrams at two levels of theory and multiple solution ion concentrations, full elemental characterization from XPS, X-ray diffraction of ZnTiN_2_ thin films following polarization, and XPS spectra for extended polarization studies (PDF)
