# Supplementary material for: Zinc Titanium Nitride Semiconductor toward Durable Photoelectrochemical Applications
Source: J Am Chem Soc. 2022 Jul 20;144(30):13673–87. doi: 10.1021/jacs.2c04241 (PMC9354241; doi:10.1021/jacs.2c04241)
Supplement: Supplementary file 1 — ja2c04241_si_001.pdf [file ja2c04241_si_001.pdf]

Electronic Supporting Information

Zinc titanium nitride semiconductor towards durable photoelectrochemical applications

Ann L. Greenaway[1]*, Sijia Ke[2,3], Theodore Culman[1], Kevin R. Talley[1], John S. Mangum[1], Karen N. Heinselman[1], Ryan S. Kingsbury[4], Rebecca W. Smaha[1], Melissa K. Gish[1], Elisa M. Miller[1], Kristin A. Persson[3,5], John M. Gregoire[6], Sage R. Bauers[1], Jeffrey B. Neaton[2,7,8], Adele C. Tamboli[1,9], Andriy Zakutayev[1]*

[1] Materials Chemical and Computational Science Directorate, National Renewable Energy Laboratory, Golden, CO 80401, USA
[2] Materials and Chemical Sciences Division, Lawrence Berkeley National Laboratory, Berkeley, CA 94720, USA
[3] Department of Materials Science and Engineering, University of California Berkeley, Berkeley, CA 94720, USA
[4] Energy Storage and Distributed Resources Division, Lawrence Berkeley National Laboratory, Berkeley, California 94720, USA
[5] Molecular Foundry, Lawrence Berkeley National Laboratory, Berkeley, California 94720, USA
[6] Division of Engineering and Applied Science, California Institute of Technology, Pasadena, CA 91125, USA
[7] Department of Physics, University of California Berkeley, Berkeley, CA 94720, USA
[8] Kavli Energy Nanosciences Institute at Berkeley, Berkeley, CA 94720, USA
[9] Department of Physics, Colorado School of Mines, Golden, Colorado 80401, USA

*Corresponding authors: ann.greenaway@nrel.gov, andriy.zakutayev@nrel.gov

$ZnTiN_2$ Crystallinity and Phase Space

$ZnTiN_2$ films were synthesized using radio-frequency co-sputtering in two separate, custom vacuum deposition systems. Initial experiments on the first co-sputtering system resulted in highly polycrystalline films (Fig. 1); annealing experiments, conducted to improve crystallinity, did not substantially change film morphology and resulted in Zn loss from previously near-stoichiometric films above 700 °C.

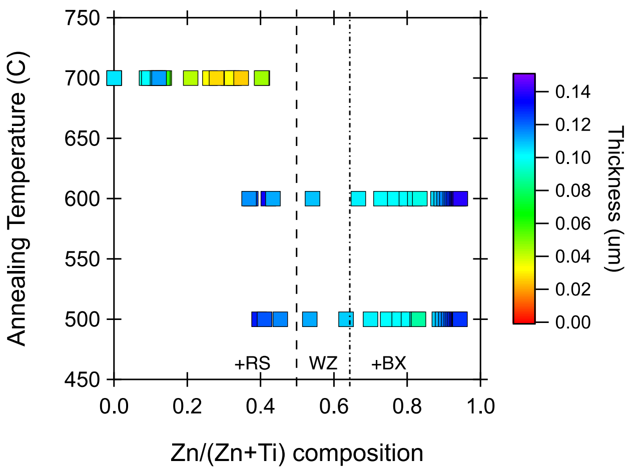

Fig. S1: Composition and thickness of polycrystalline Zn-Ti-N films following rapid thermal annealing. Significant Zn-N loss and thickness reduction is observed at and above 700 °C.

Optimization of $ZnTiN_2$ film crystallinity (Section 2.2) was performed on the second deposition system, using substrates with intentional temperature gradients during film deposition. Temperature gradients are introduced across the substrate using a custom holder that contacts only one side of the substrate (approximately 12 mm from the edge) while suspending the remainder of the substrate in vacuum such that it does not contact the stage. When the stage is heated from the back side, the part of the substrate contacting the stage will heat up, but the opposite side will remain cold, with the temperature gradient determined by the thermal diffusivity of the substrate. The temperature gradient is calibrated for a particular substrate material by attaching thermocouples to both the "hot" and "cold" and measuring the respective temperatures over a range of heater setpoints (0 °C to 1000 °C) in intervals of 100 °C. Polynomial fits are made to the resulting hot/cold data and used to calculate the temperature gradient at any heater setpoint within the calibrated range with reasonable accuracy. Grazing-incidence x-ray diffraction was performed on films with optimized crystallinity to confirm that the previously observed structure of $ZnTiN_2$ was maintained (Fig. S2).

Table S1: Deposition parameters for Zn-Ti-N combinatorial libraries shown in Fig. 4a. Parameters kept constant across all depositions were: N_2 flow rate = 50 sccm; Ar flow rate = 100 sccm; target-to-substrate distance = 190 mm; deposition time = 120 min.

Sample Name	Temperature Gradient (Hot Side – Cold Side) (°C)	Zn Power (W)	Ti Power (W)	Chamber Pressure (mTorr)
C9_2	200 – 60	25	100	6
C9_3	200 – 60	20	150	3.5
C9_4	200 – 60	10	150	3.5
C9_5	300 – 80	15	150	3.5
C9_6	300 – 80	20	150	3.5

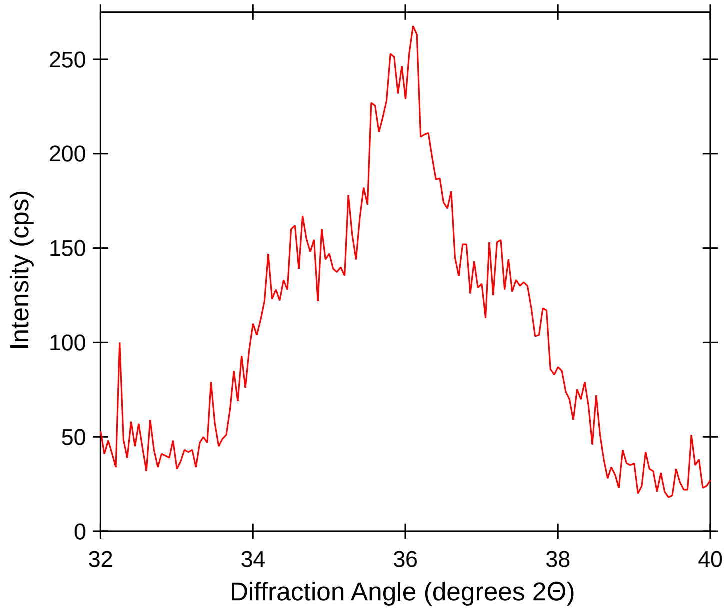

Fig. S2: Grazing-incidence XRD on highly-(002) textured $ZnTiN_2$ film, showing the (100), (002), and (101) wurtzite peaks identified in **Fig. 1a**.

$ZnTiN_2$ Optical Properties

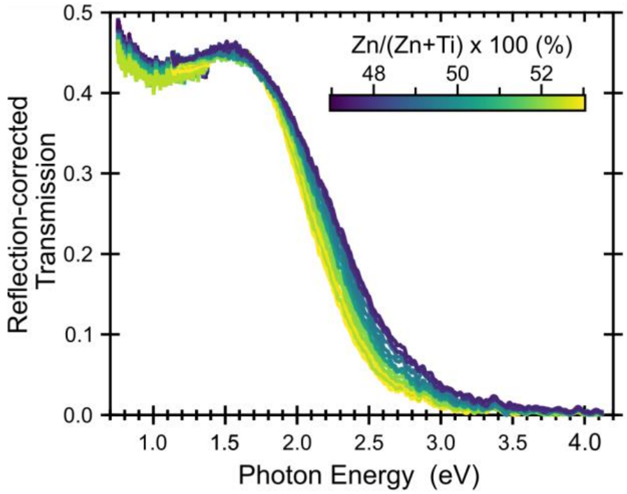

Fig. S3: Reflection-corrected UV-vis-NIR transmission data for $ZnTiN_2$ thin films

Transient absorption spectroscopy was used to measure the photoresponse of the $ZnTiN_2$ thin film library shown in Fig. 6a at three different Zn/(Zn+Ti) compositions. The fit kinetics of the films at 600 nm is shown in Fig. 6b in the main text, and the time constants for each fit are given here in Table S2. A multiexponential fit best describes the data due to the high defect density in this system. The offset, y_0, shows the percentage of carriers remaining with lifetimes longer than 5 ns. This percentage decreases with increasing Zn concentration with 53% Zn/(Zn+Ti) having the fewest long-lived carriers, in agreement with resistivity measurements. Each amplitude (A_x) refers to the percentage of carriers lost within the corresponding time constant. For all Zn/(Zn+Ti) compositions, the largest loss of free carriers occurs within 1 ps after photoexcitation. Fig. S4a shows the spectra across energies of the Zn/(Zn+Ti) = 50% thin film at multiple times following excitation. Fig. S4b compares the spectra of the three samples at 0.25 ps, and shows the same trend in absorption shift with composition as in Fig. 6a.

Table S2 Fit decay kinetics for the three $ZnTiN_2$ thin film compositions shown in Fig. 6c.

	**Zn/(Zn+Ti) (%)**		
	**47**	**50**	**53**
**y_0**	33%	22%	14.2%
**A_1**	36%	47.3%	58.6%
**τ_1, ps**	0.58 (±0.14)	0.48 (±0.06)	0.64 (±0.05)
**A_2**	25%	25.4%	19.8%
**τ_2, ps**	3.9 (±0.9)	3.9 (±0.4)	8.7 (±1.4)
**A_3**	6.4%	6.2%	7.3%
**τ_3, ps**	625 (±174)	207 (±39)	403 (±103)

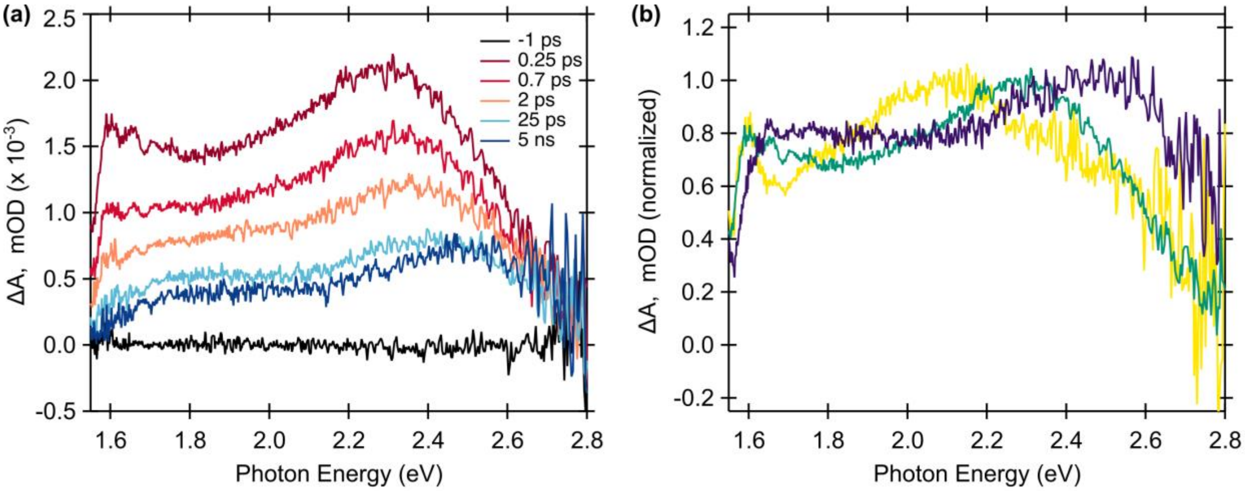

Fig. S4: (a) Full transient absorption spectra for Zn/(Zn+Ti) = 50% thin film (b) Normalized absorption spectra at 0.25 ps for Zn/(Zn+Ti) = 47% (purple), 50% (teal), and 53% (yellow) after 3.1 eV photoexcitation, showing the same trend in absorption (peak) shift as Fig. 6.

Computational and Experimental Electrochemical Stability

The Pourbaix diagram for $ZnTiN_2$ presented in Fig.9a is constructed from a combination of metaGGA and GGA density functional theory (DFT) calculations[1] using the Materials Project DFT mixing scheme.[2] Fig. S5 compares the solid stable phases predicted by the pure PBE diagram to those predicted by the mixed (r^2SCAN + PBE) diagram. Although the stable solid phases have slightly different bounds, they are broadly similar across the two levels of theory at the same concentrations.

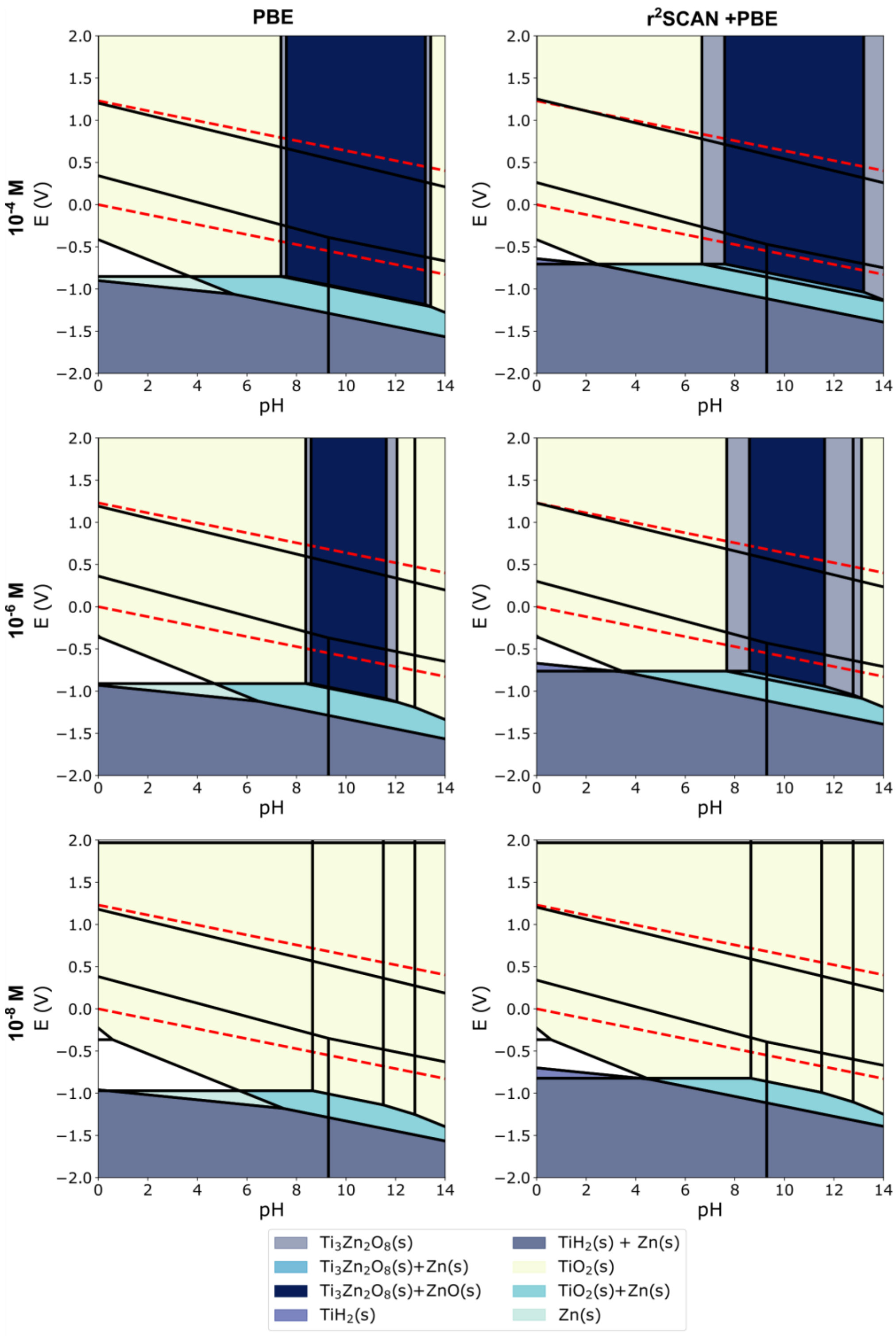

Fig. S5: Pourbaix diagrams showing stable solid phases for $ZnTiN_2$ in aqueous solution, calculated from Materials Project PBE GGA calculations (left) and a combination of r^2SCAN metaGGA and PBE GGA calculations using the Materials Project DFT mixing scheme (right; see Methods) for 1:1:2 ratios of Zn:Ti:N at ionic concentrations of 10^{-4} M, 10^{-6} M, 10^{-8} M. Dotted red lines indicate the oxygen evolution reaction (top) and hydrogen evolution reaction (bottom) potentials in each diagram

Table S3: Full elemental percentages for XPS analysis of $ZnTiN_2$ electrodes including post-electrochemistry contamination from supporting electrolyte. For each pH, the pre-electrochemistry composition of the film is shown for ease of comparison to the post-electrochemical (echem) measurements. Compositions are +/− 5%.

		$ZnTiN_2$ components only (normalized to 100%)			All observed elements (sum to 100%)							
		**Zn**	**Ti**	**N**	**Zn**	**Ti**	**N**	**O**	**C**	**Na**	**K**	**P**
**pH 5**	**Pre-echem**	86.8	5.02	7.70	55.51	3.19	4.9	25.0	11.4	--	--	--
	**−0.2 V vs RHE**	27.2	31.5	41.2	9.28	10.74	14.04	39.45	16.25	5.6	1.78	2.85
	**+0.5 V vs RHE**	27.2	31.7	41.0	12.94	15.05	19.48	36.29	3.24	8.04	1.67	3.3
	**−0.2 V vs RHE (45 min)**	13.2	39.1	47.7	4.15	12.37	15.05	41.56	18.89	1.17	2.26	4.55
**pH 9**	**Pre-echem**	86.9	5.15	7.91	55.18	3.3	5.0	24.7	11.8	--	--	--
	**−0.2 V vs RHE**	51.8	17.5	30.7	25.51	8.62	15.09	25.29	13.76	11.73	0	0
	**+0.5 V vs RHE**	56.6	15.7	27.6	26.19	7.28	12.82	25.74	15.18	12.79	0	0
	**−0.2 V vs RHE (45 min)**	24.6	30.5	44.9	10.78	13.38	19.73	29.13	22.34	4.64	0	0
**pH 11**	**Pre-echem**	85.6	5.54	8.84	48.53	3.14	5.0	26.9	16.4	--	--	--
	**−0.2 V vs RHE**	47.2	20.5	32.3	4.96	2.16	3.4	37.6	8.67	39.17	0	4.03
	**+0.5 V vs RHE**	48.3	20.1	31.6	9.2	3.82	6.03	35.39	8.03	34.26	0.02	3.26

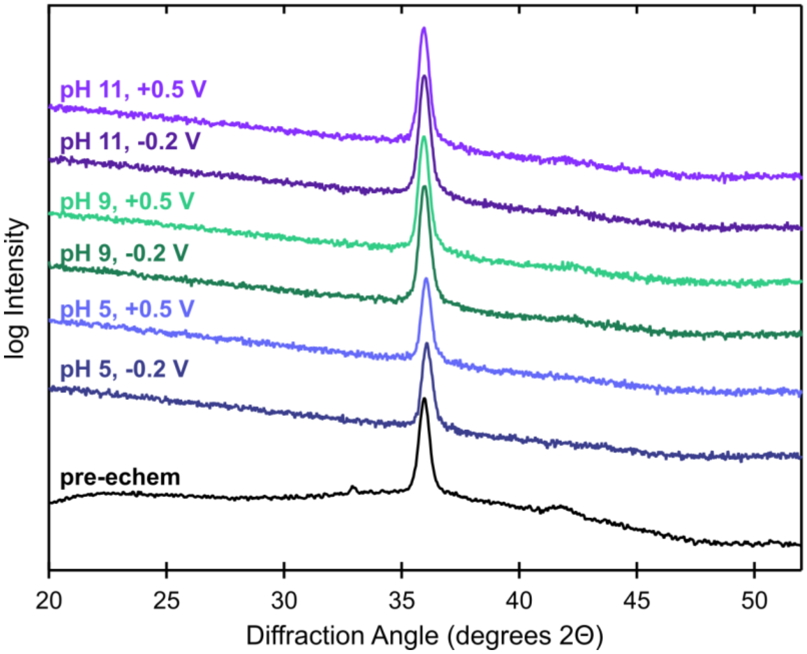

Fig. S6: Post-XPS XRD (Cu Kα) for each of the $ZnTiN_2$ electrodes in **Fig. 9** (15-minute polarizations only), showing no new phases resulting from polarization. The slight peak shift between the pre-echem and polarized traces is a result of differences in alignment.

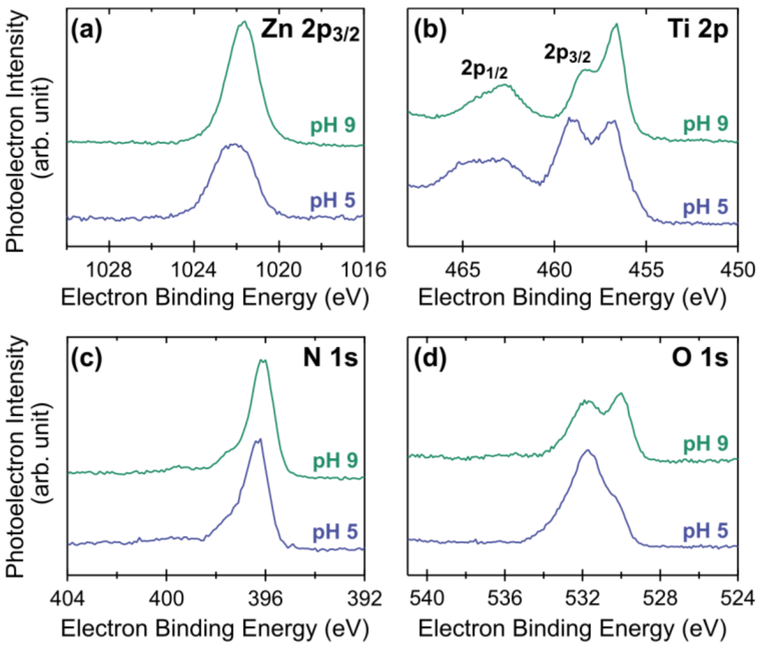

Fig. S7: XPS spectra for individual elements in $ZnTiN_2$ thin films following forty-five minute polarization studies (labeled with pH) at −0.2V vs RHE. (a) Zn $2p_{3/2}$ (b) Ti 2p (c) N 1s (d) O 1s. In all cases, the spectra are qualitatively similar to those observed after fifteen-minute polarizations, indicating the formation of distinct oxide environments at pH 5 and pH 9.

References

(1) Persson, K. A.; Waldwick, B.; Lazic, P.; Ceder, G. Prediction of Solid-Aqueous Equilibria: Scheme to Combine First-Principles Calculations of Solids with Experimental Aqueous States. *Phys. Rev. B* **2012**, *85* (23), 235438. https://doi.org/10.1103/PhysRevB.85.235438.

(2) Kingsbury, R.; Rosen, A. S.; Gupta, A. S.; Munro, J.; Ong, S. P.; Jain, A.; Dwaraknath, S.; Horton, M. K.; Persson, K. A. A Flexible and Scalable Scheme for Mixing Computed Formation Energies from Different Levels of Theory. **2022**. https://doi.org/10.26434/chemrxiv-2022-3ptwx.
